# Crossing fitness valleys via double substitutions within codons

**DOI:** 10.1186/s12915-019-0727-4

**Published:** 2019-12-16

**Authors:** Frida Belinky, Itamar Sela, Igor B. Rogozin, Eugene V. Koonin

**Affiliations:** National Center for Biotechnology Information, National Library of Medicine, National Institutes of Health, Bethesda, MD USA

**Keywords:** Natural selection, Bacteria, Archaea, Short-term evolution, DNA context, Double substitutions

## Abstract

**Background:**

Single nucleotide substitutions in protein-coding genes can be divided into synonymous (S), with little fitness effect, and non-synonymous (N) ones that alter amino acids and thus generally have a greater effect. Most of the N substitutions are affected by purifying selection that eliminates them from evolving populations. However, additional mutations of nearby bases potentially could alleviate the deleterious effect of single substitutions, making them subject to positive selection. To elucidate the effects of selection on double substitutions in all codons, it is critical to differentiate selection from mutational biases.

**Results:**

We addressed the evolutionary regimes of within-codon double substitutions in 37 groups of closely related prokaryotic genomes from diverse phyla by comparing the fractions of double substitutions within codons to those of the equivalent double S substitutions in adjacent codons. Under the assumption that substitutions occur one at a time, all within-codon double substitutions can be represented as “ancestral-intermediate-final” sequences (where “intermediate” refers to the first single substitution and “final” refers to the second substitution) and can be partitioned into four classes: (1) SS, S intermediate–S final; (2) SN, S intermediate–N final; (3) NS, N intermediate–S final; and (4) NN, N intermediate–N final. We found that the selective pressure on the second substitution markedly differs among these classes of double substitutions. Analogous to single S (synonymous) substitutions, SS double substitutions evolve neutrally, whereas analogous to single N (non-synonymous) substitutions, SN double substitutions are subject to purifying selection. In contrast, NS show positive selection on the second step because the original amino acid is recovered. The NN double substitutions are heterogeneous and can be subject to either purifying or positive selection, or evolve neutrally, depending on the amino acid similarity between the final or intermediate and the ancestral states.

**Conclusions:**

The results of the present, comprehensive analysis of the evolutionary landscape of within-codon double substitutions reaffirm the largely conservative regime of protein evolution. However, the second step of a double substitution can be subject to positive selection when the first step is deleterious. Such positive selection can result in frequent crossing of valleys on the fitness landscape.

## Background

In classic population genetics, mutations are usually assumed to occur one at a time, independently of each other [1–5]. However, clustering of mutations and substitutions (i.e., mutations that have been fixed in the population), in particular, those occurring in adjacent sites (multi-nucleotide mutations/substitutions), has been documented in many diverse organisms [6–13]. Multi-nucleotide substitutions potentially could originate from mutational biases, selection, or a combination of both. Recently, it has been claimed that positive selection is overestimated by the branch-site test (BST) because many if not most of the sites supporting positive selection actually are multi-nucleotide substitutions that could result from multi-nucleotide mutations [14]. However, independent of BST, double substitutions within the same codon in protein-coding genes have been repeatedly claimed to be driven by positive selection. This conclusion follows from the comparison of the observed frequencies of double substitutions to those expected from the frequencies of single substitutions. If the frequency of a double substitution is significantly greater than the product of the frequencies of the respective single substitutions, positive selection is inferred [15–17]. Such apparent signs of positive selection affecting double substitutions have been detected as a general trend in the mouse-rat lineage [15]. Similar conclusions have been reached for double substitutions in codons for serine, the only amino acid that is encoded by two disjoint series of codons. In the case of serine, the proposed scenario is that a non-synonymous (N) substitution that leads to the replacement of a serine with another amino acid and is hence deleterious is followed by a second substitution that restores serine and, accordingly, the protein function and the original fitness value [17]. The fixation of the second mutation has been attributed to positive selection, and the observed excessive frequency of double substitutions has been explained by this effect of selection, as opposed to a mutational bias.

Similarly, signatures of positive selection have been found for double substitutions in stop codons in bacteria (UAG > UGA and UGA > UAG), which could be attributed to the deleterious, non-stop intermediate state, UGG [16]. Furthermore, slightly advantageous back mutations are expected under the nearly neutral model [18]. Thus, a second mutation in a codon that reverts a deleterious non-synonymous substitution to restore the codon for the original amino acid is expected to be advantageous. However, given that the apparent positive selection in codon double substitutions could be potentially explained by biased mutational processes that favor multi-nucleotide mutations [6, 9, 14, 17, 19], it is essential to compare codon double substitutions to an appropriate null model in order to accurately infer selection.

When two (or more) nucleotide substitutions are observed in a codon or in a null model, it is not known whether such an observation results from two independent single nucleotide mutational events or whether it is the result of one multi-nucleotide mutation (i.e., a simultaneous change of more than one nucleotide). Therefore, we collectively refer to observations with two nucleotide differences (compared to the ancestral state) as double substitutions which include both sequential single mutations where each was fixed to become a substitution independently, and simultaneous multi-nucleotide mutations that were fixed together as one unit (and are referred to explicitly when we present the corresponding estimates). Following the well-established principles of identification of selective pressure by comparison of non-synonymous to synonymous rates [20–26], to assess the selection that affects double substitutions within codons, we compared the fractions of non-synonymous double substitutions within codons to the respective fractions for adjacent, equivalent double synonymous substitutions. We categorize codon double substitution into four classes and show that these classes of codon double substitution are associated with different types of selection acting on the second substitution step (final outcome of the double substitution compared to the initial state).

## Results

### Inference of selection on codon double substitutions by comparison to null models

**Fig. 1:**
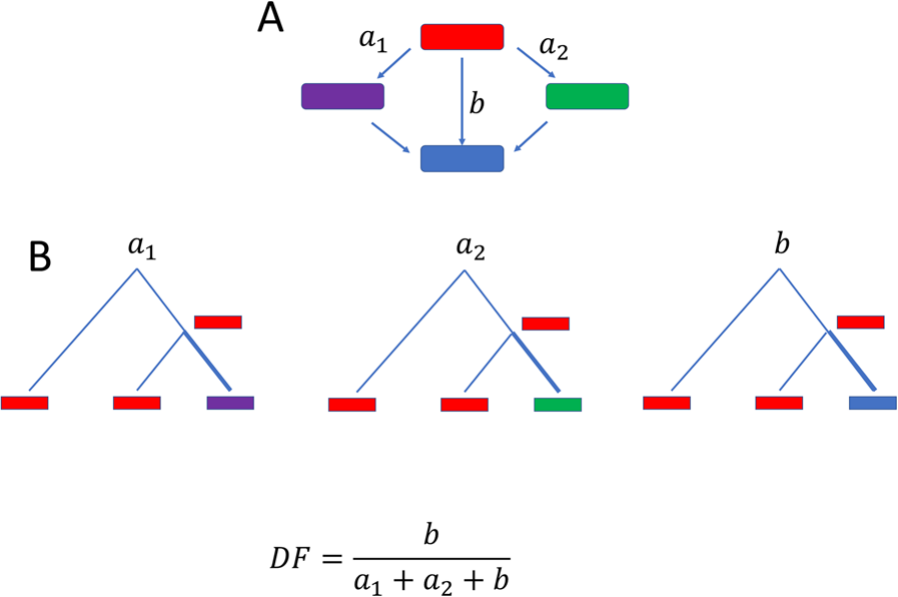
Conceptual scheme of double substitution analysis and the double fraction (DF) measure. **a** Point mutations are assumed to appear one at a time, such that observed double substitutions (*b*) occur through intermediate single substitution states. For each double substitution instance, there are two possible single substitution trajectories (*a*_1_ and *a*_2_). **b** Instances of single or double substitutions are inferred from the genomic data by construction of genomes triplets and relying on parsimony principle (see the “Methods” section). In brief, the parsimony principle implies that mutations occur along the thick branches in the trees shown in **b**. The double fraction is defined as the ratio between the number of double substitution instances *b* and the sum of relevant single (*a*_1_ + *a*_2_) and double (*b*) substitution instances

**Fig. 2:**
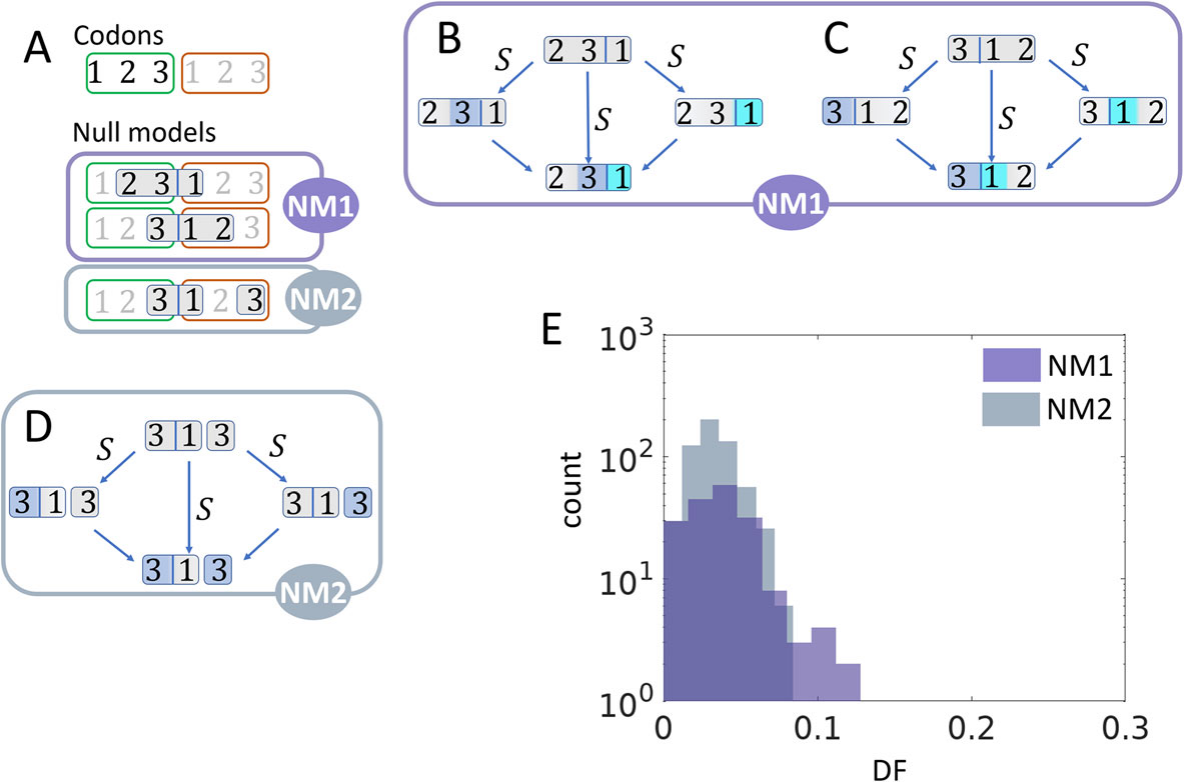
Null models that are used to infer selection on double substitutions through the DF measure. **a** The selection on double substitutions is inferred by comparing the DF for codons and their respective null models (NM1 and NM2). Two adjacent codons are illustrated, and the nucleotide position within the codon is indicated, according to the reading frame. The two null models are artificial codons constructed by considering positions from two adjacent codons, denoted codon *i* (indicated in green) and codon *ii* (indicated in orange). **b** The first configuration of the null model NM1. A constant second codon position in codon *i*, followed by a fourfold degenerate site in the third codon position of codon *i* which is followed by a twofold degenerate site in the first position of codon *ii*. All substitutions are synonymous (S). Substitution in a fourfold degenerate site is indicated by blue shading of the mutated codon position, and cyan shading indicates a substitution in a twofold degenerate site. **c** The second configuration of the null model NM1. A fourfold degenerate site in the third codon *i* position followed by a twofold degenerate site in the first position of codon *ii*, which is followed by a constant base in the second codon position of codon *ii*. All substitutions are synonymous (S). Substitution in a fourfold degenerate site is indicated by blue shading of the mutated codon position, and cyan shading indicates a substitution in a twofold degenerate site. **d** Null model NM2. A fourfold degenerate site in the third position of codon *i* followed by a constant first codon position in codon *ii* and by a fourfold degenerate site in the third codon *ii* position (skipping the second position of codon *ii*). All substitutions are synonymous (S), and substitutions in the fourfold degenerate sites are indicated by blue shading of the mutated codon position. **e** Comparison of DF between the two null models, NM1 (adjacent synonymous substitutions) as in **b** and **c** and NM2 (non-adjacent synonymous substitutions) as in **d**. The difference between the two distributions is significant according to *t* test (*p* value = 0.0038) but not significant under the *U* test (*p* value = 0.104)

From 37 triplets of genomes with reliable phylogenetic relationships that were extracted from the Alignable Tight Genomic Clusters (ATGC) database [17, 27], we obtained counts of double and single substitutions within codons, and in double synonymous controls based on the parsimony principle (Fig. 1; see the “Methods” section for details of the analyzed dataset). We use the double fraction (DF) as a measure of selection (Fig. 1). The DF is calculated as the observed double substitution count divided by the sum of the single and double substitution counts. The selection on double substitutions was inferred, primarily, by comparing the DF for within-codon non-synonymous substitutions to two null models. This approach is analogous to the traditional estimation of the strength of selection for single substitutions, where the rate of non-synonymous substitutions is compared to that of synonymous substitutions which are assumed to be neutral and, accordingly, are used as the null model [20–26]. The key difference between the present work and the previous studies on double substitutions [15, 28, 29] is that here, all the analyses included comparison to double synonymous substitutions, adjacent and non-adjacent, that serve as null models for the double substitutions in codons. Although previous studies have used double synonymous substitutions as null models [15, 28, 29], here, we account for the first time for the rate of adjacent double synonymous substitutions with the same nucleotide changes as the corresponding within-codon double substitutions. Such double synonymous substitutions are explicitly used to control for the occurrence of multi-nucleotide substitutions which is critical given the wide spread of context biases of mutation rates [30–32]. Although it is well known that transition and transversion rates differ substantially [22, 33, 34], it is unclear to what extent the adjacency of mutations is affected by base composition. For example, DNA polymerase η tends to produce an excessive amount of simultaneous double transitions in A/T-rich context [31] whereas DNA polymerase ζ frequently produces transversions in C/G-rich context [30, 32]. Another important issue is the balance between consecutive double substitutions (independent stepwise fixation of adjacent mutations) and simultaneous double substitutions. This problem cannot be ignored because some replication enzymes are known to produce or initiate production of excessive amounts of simultaneous double substitutions under certain conditions [35–39]. Therefore, we compared the DF values of all codon double substitutions to all possible types of double synonymous substitutions that were captured in the two null models (Fig. 2).

The null models were constructed from codon-like 3-base sequences from two neighboring codons, as illustrated in Fig. 2a. Specifically, three configurations that are shown in Fig. 2b–d were collected from the same data.

The first null model (NM1) included a synonymous substitution in the third position of a codon followed by another synonymous substitution in the first position of the next codon. The second null model (NM2) included non-adjacent synonymous substitutions in third codon positions of consecutive codons. We found that the double fraction (DF) was typically higher for the NM1 model compared to the NM2 model suggesting the existence of a mutational bias in adjacent positions (Fig. 2e). This difference was only statistically significant under the *t* test but not with the non-parametric *U* test. The difference between NM1 and NM2 is that whereas NM1 includes adjacent double synonymous substitutions, the double synonymous substitutions in NM2 are two bases apart. Thus, NM1 is a better control for adjacent codon double substitutions, and conversely, NM2 is preferable for non-adjacent codon double substitutions, i.e., substitutions in positions 1 and 3 in the codon. As expected from previous findings, the DF value for NM1 is greater than the one for NM2 because of the excess of multi-nucleotide mutations at adjacent sites.

The DF is assumed to be mostly affected by the substitution rate at the second step although some effect of the first step rate cannot be ruled out. If the elevated DF of codon double substitutions results solely from a multi-nucleotide mutational bias, the comparison to the null model is expected to show no significant difference (after removing the estimated number of substitutions arising from double mutations—see the “Methods” section). Conversely, a significantly lower DF compared to that of the null model is indicative of purifying selection, whereas a significantly higher DF points to positive selection (see the “Methods” section for further detail).

In addition to the primary comparison of each of the group DF values to those of the null models, we performed two complementary analyses to further test for selection. First, each individual codon double substitution was compared to a particular null model with the same base changes, thus accounting for the nucleotide context effects. Second, we compared the DF between fast and slow evolving genes. The rationale behind this analysis is that slow evolving genes have fewer changes to their amino acid sequences and as a result have a lower *dN/dS* compared to fast evolving genes (by definition; see the “Methods” section for detail). Thus, changes that affect the protein structure and functionality, i.e., non-synonymous substitutions, occur more frequently in fast evolving than in slow evolving genes (Additional file 1: Figure S1) whereas synonymous substitutions occur at nearly equal rates in fast and in slow evolving genes (Additional file 1: Figure S1), supporting the notion that they are nearly neutral. In the same vein, under the assumption that selection on a gene as a whole is a major determinant of the selection on a substitution at hand, double substitutions that are subject to purifying selection are expected to have higher DF in fast vs. slow evolving genes, and conversely, changes driven by positive selection would have higher DF in slow vs. fast evolving genes. Indeed, in the latter case, positive selection is associated with those substitutions (second in the double combination) that restore the original amino acid (or its properties in the case of a conservative replacement). This selection is expected to be stronger in slow evolving genes that are, overall, subject to strong purifying selection.

### Distinct selection regimes for different types of codon double substitutions

**Fig. 3:**
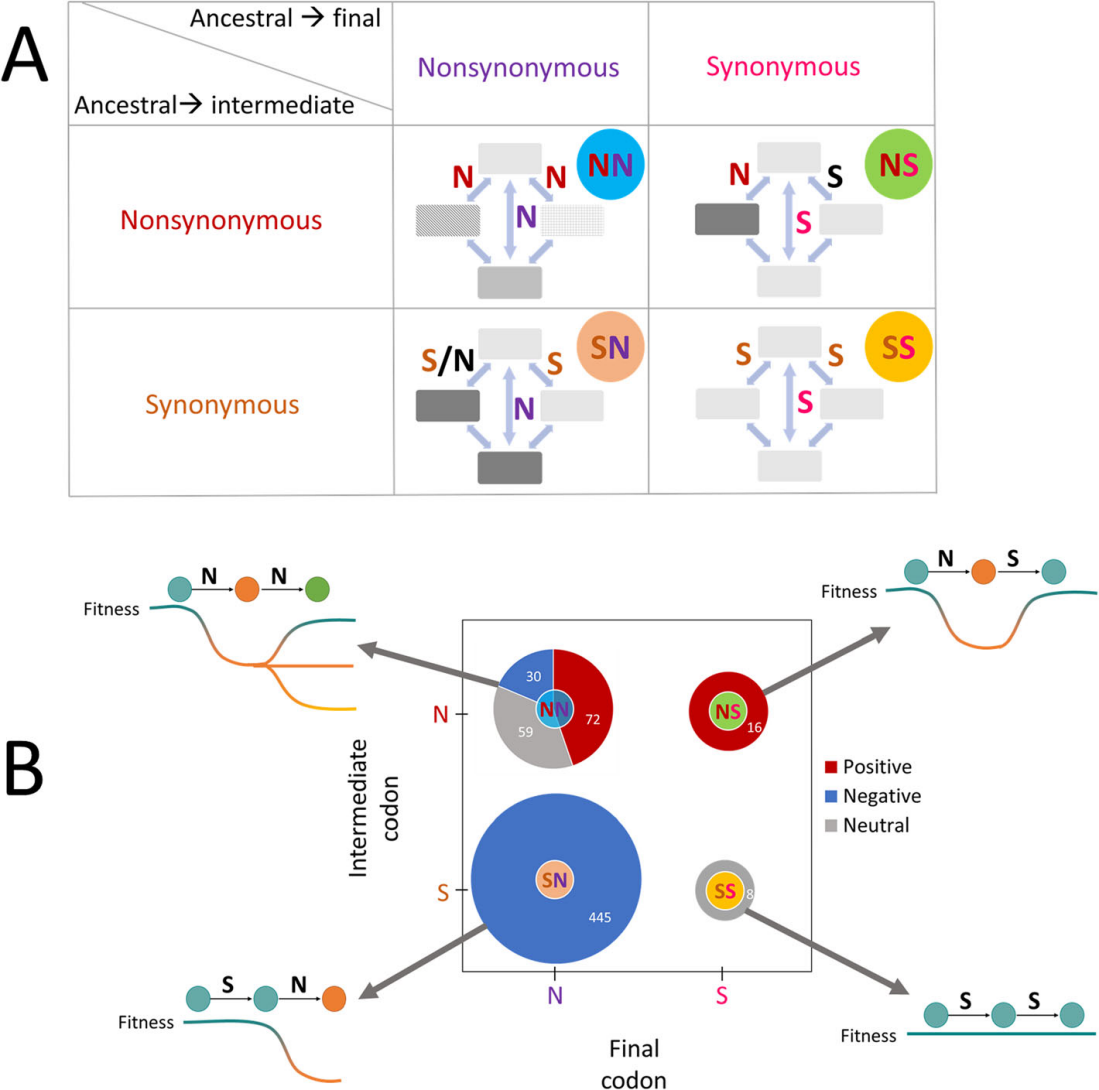
Classification of the double codon substitutions. **a** Four combinations of within-codon double substitutions based on the synonymy of the respective ancestral and derived (final) codons, and synonymy of intermediate state codons to the ancestral codons. Each cell represents one of the four combinations of the two intermediates (non) synonymy and the two final states (non) synonymy. When both are synonymous, the combination is noted as SS. When at least one of the intermediates is synonymous to the ancestral codon, whereas the final codon is non-synonymous to the ancestral state, the combination is classified as SN. When one of the intermediates is non-synonymous to the ancestral codon, whereas the final codon is synonymous to the ancestral, the combination is classified as NS. Finally, when both are non-synonymous, the combination is noted as NN. The text colors represent (non) synonymy of the intermediate and final codons compared to the ancestral: brown, synonymous intermediate; red, non-synonymous intermediate; pink, synonymous final; purple, non-synonymous final. The circle colors are different for each class of codon double substitutions and are the same as in other figures: yellow, SS; light orange, SN; light green, NS; and light blue, NN. **b** Selective pressure in different codon double substitutions classes. *Positive*, combinations compatible with positive selection, where a codon double substitution has a significantly higher DF than the corresponding double synonymous substitution and the DF is lower in fast compared to slow evolving genes. *Negative*, combinations compatible with purifying selection, where a codon double substitution has a significantly lower DF than the corresponding double synonymous substitution and the DF is higher in fast compared to slow evolving genes. *Neutral*, combinations where the codon DF was not significantly different from that of the corresponding synonymous DF and the DF is similar in fast and slow evolving genes. All SS, SN, and NS combinations show compatible results in the comparison of the DF to the double synonymous null models, and in the comparison of the DF between fast and slow evolving genes, and thus are collectively presented as being subject to neutral evolution, negative and positive selection, respectively. However, the NN combinations show conflicting results between the comparison of DF to double synonymous control null models and the comparison of DF between fast and slow evolving genes, and are therefore presented as a combination of positive, negative, and neutral regimes, based on the individual comparisons to the specific null models with the same base composition

Representing all within-codon double substitutions in the general form, “ancestral-intermediate-final,” we define the following four classes of double substitutions where S and N stand for synonymous and non-synonymous (respectively), with respect to the ancestral codon (Fig. 3a):

1. SS—both intermediate single substitutions are synonymous, and the double substitution is also synonymous.
2. SN—at least one of the intermediate single substitutions is synonymous, where the other single substitution can be either synonymous or non-synonymous. The double substitution is non-synonymous to the ancestral state.
3. NS—one of the intermediate single substitutions is synonymous, and the other non-synonymous. The double substitution is synonymous to the ancestral state.
4. NN—both intermediate single substitutions are non-synonymous, and the double substitution is non-synonymous to the ancestral state.

The special case where both intermediate single substitutions are non-synonymous, but the double substitution is synonymous to the ancestral state occurs only in double substitutions between the two series of serine codons. This special case has been analyzed in detail elsewhere [17]. For the sake of completeness, the current analysis was also applied to the serine case (Additional file 1: Figures S2–S3).

The results of the three analyses reveal distinct selection regimes for the four classes of codon double substitutions (Fig. 3b). Like single synonymous changes, SS double synonymous are also nearly neutral as they have almost the same DF values in fast and slow evolving genes, and neutrality cannot be rejected; the SN changes where one of the intermediates is synonymous while the final codon is non-synonymous (analogous to single non-synonymous substitutions) are subject to purifying selection. The higher DF in NS double substitutions in slow genes compared to fast is consistent with positive selection. If NS changes were not positively selected, it is more likely to observe a similar DF in fast and slow evolving genes, but this is not the case. Finally, NN changes exhibit a mixture of all three regimes depending on the similarity of the amino acids encoded by the intermediate and final codons to the original amino acid.

### NS: non-synonymous substitution followed by recovery of the ancestral amino acid

**Fig. 4:**
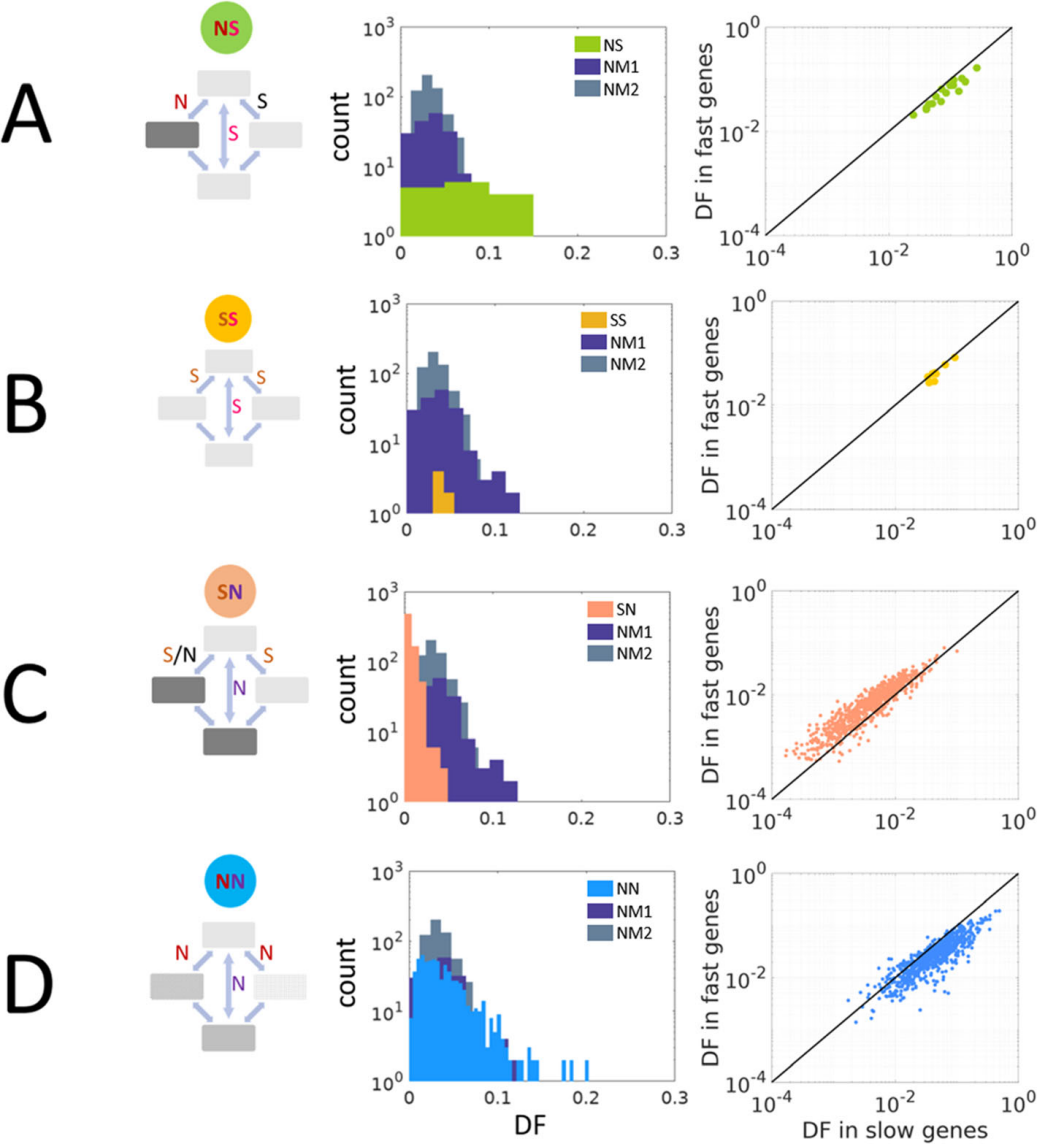
Selective regimes of the codon double substitutions. The panels on the left show the comparison of each codon double substitution class to the double synonymous null models, and the panels to the right show the comparisons between the DF of each of the classes in fast vs. slow evolving genes. Purple, NM1; gray, NM2; light green, NS; yellow, SS; light orange, SN; light blue, NN. **b** NS, one non-synonymous intermediate, synonymous final codon. *t* test with NM1 *p* value = 1.92 × 10^−09^, *U* test with NM1 *p* value = 4.40 × 10^−05^, *t* test with NM2 *p* value = 1.11 × 10^−27^, *U* test with NM2 *p* value = 1.12 × 10^−06^. **b** SS, double synonymous codon substitutions. *t* test with NM1 *p* value = 0.25, *U* test with NM1 *p* value = 0.13, *t* test with NM2 *p* value = 0.007, *U* test with NM2 *p* value = 0.02. **c** SN, at least one synonymous intermediate codon, non-synonymous final codon. *t* test with NM1 *p* value = 9.73 × 10^−127^, *U* test with NM1 *p* value = 8.17 × 10^−70^, *t* test with NM2 *p* value = 7.72 × 10^−232^, *U* test with NM2 *p* value = 2.7 × 10^−177^. **d** NN, both intermediates and the final codon are non-synonymous to the ancestral. *t* test with NM1 *p* value = 0.14, *U* test with NM1 *p* value = 0.94, *t* test with NM2 *p* value = 1.45 × 10^−07^, *U* test with NM2 *p* value = 0.19

The NS double substitutions show significantly higher DF values compared to both null models (Fig. 4a). This pattern is compatible with positive selection driving the second substitution which returns to the original amino acid state. The NS double substitutions also showed higher DF in slow compared to fast evolving genes although the observed frequencies of double substitutions were similar, which is compatible with positive selection (Fig. 4a). Analysis of individual NS double substitutions (Additional file 2), after the Benjamini–Hochberg (BH) correction, resulted in the detection of significant positive selection for 15 of the 16 combinations (93%). Of the 11 combinations for which the DF values could be reliably compared to those under the null model (see the “Methods” section), 10 exhibited positive selection (91%). The only exception is TTG > CTC, for which the DF of the NS change was greater than that of the null model, but the difference was not statistically significant. The observed frequency of NS double substitutions is close to that in the null model (Additional file 1: Figure S4B) suggesting that the overall selection is about the same as that on double synonymous substitutions. However, negative selection on the non-synonymous intermediate (Additional file 1: Figure S4D), if followed by a neutral substitution, would result in an overall lower frequency of NS double substitution compared to the null model. Comparison of the expected NS frequencies under assumed neutrality of the second substitution confirms that for all NS combinations, these frequencies are significantly lower than the corresponding values for the null model. By contrast, the observed frequencies of all 16 NS combinations are either not significantly different compared to the observed null model values (5 combinations) or are significantly higher (11 combinations) (Additional file 1: Table S1). The higher (and even similar) observed frequencies of NS double substitutions compared to the null model, together with the finding that DF values are higher in NS combinations compared to the null model, together, point to dominant positive selection in the NS class.

### SS: double synonymous substitutions

For double synonymous substitutions, neutrality cannot be rejected by comparison to both null models using the *U* test (the appropriate test for this small sample size; Fig. 4b). Thus, the DF values of the SS substitutions can be explained by the frequency of multi-nucleotide mutations suggesting that SS double substitutions evolve (nearly) neutrally, similar to single synonymous substitutions, as evident by their similar rates in fast and slow evolving genes (Additional file 1: Figure S1). Additionally, there was no significant difference between the DF values of SS double substitutions in fast and slow evolving genes (Fig. 4b) which is compatible with the neutral evolutionary regime. Nevertheless, when comparing individual SS combinations to their respective null models with the same base changes (Additional file 2), weak positive selection was detected after the BH correction for most of these double substitutions. This weak selection can be linked to codon bias (Additional file 1: Figure S5). However, the small number of SS combinations [8] gives one limited power at best to draw strong conclusions regarding selection on the entire group. Furthermore, the SS class could, in principle, serve as a null model as these combinations are also double synonymous substitutions (see the “Methods” section for limitations of the use of SS as a null model).

### SN: synonymous substitution followed by a non-synonymous one

The DF values for SN double substitutions are significantly lower than those for both NM1 and NM2 null models (Fig. 4c), indicating that the second step of these double substitutions is subject to purifying selection. Similarly to single non-synonymous substitutions (Additional file 1: Figure S1), the SN doubles show significantly higher DF values in fast evolving genes compared to slow evolving genes (Fig. 4c), which is also indicative of purifying selection. Analysis of individual combinations of SN substitutions with the same base changes (Additional file 2), after the BH correction, showed that 88% were compatible with purifying selection, for 10% neutrality could not be rejected, and less than 2% were compatible with positive selection (Additional file 2).

### NN: double non-synonymous substitutions

For the NN double substitutions, detailed comparison of individual combinations reveals a mixture of positive selection, purifying selection, and neutral evolution. Neutrality can be rejected by comparison of the DF values of the NN doubles to the NM2 null model but not to the NM1 null model (Fig. 4d). In contrast, the comparison between slow and fast evolving genes shows that the DF value of NN doubles is higher in slow compared to fast evolving genes, which is compatible with positive selection. Given this discrepancy between the results of the group level DF analysis and the comparison between fast and slow evolving genes (Fig. 4D), the most likely explanation is a mixture of selection regimes, as shown by the individual comparisons for each NN combination (Additional file 1: Table S2). After BH correction for multiple testing, we found evidence of positive selection for 44% of the NN doubles, purifying selection for 19%, and neutral evolution for 36% (Fig. 3b, Additional file 2).

In a unique subset of the NN substitutions (eight combinations), the first substitution is non-synonymous whereas the second one is synonymous (for example, TTT->CTG). In these NN combinations, the intermediate amino acid does not change following the second substitution, and therefore, the second substitution in this subclass is expected to be neutral. Indeed, in five of the eight such NN combinations, where both intermediates are synonymous to the final codon, but non-synonymous to the ancestral codon, neutrality could not be rejected (see Additional file 2). The three combinations for which neutrality could be rejected (see Additional file 2) are compatible with positive selection and might reflect selection for codon choice bias as also observed in some of the SS combinations.

### Modes of selection reflect amino acid similarity

**Fig. 5:**
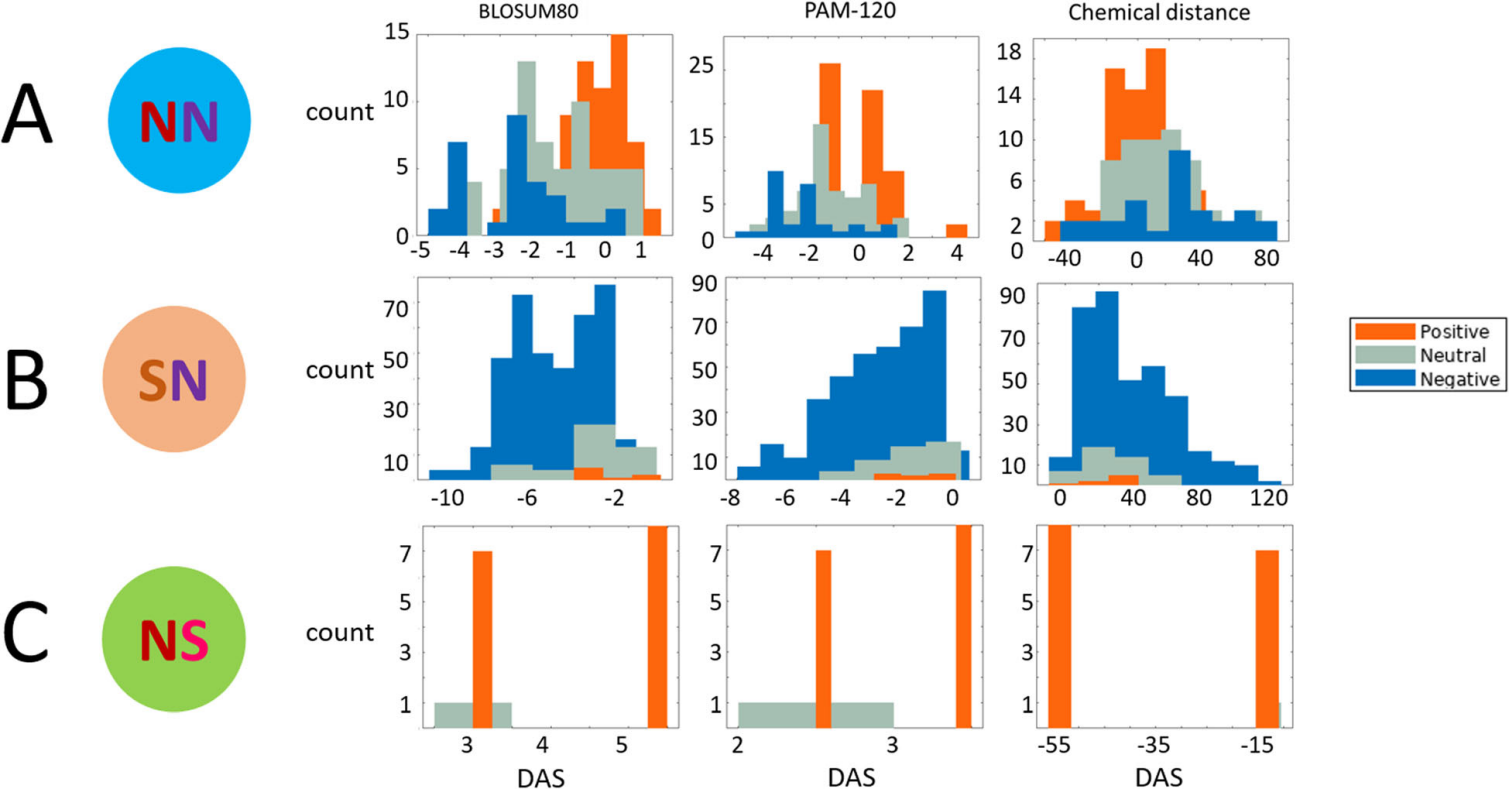
Similarity between the ancestral, intermediate, and final amino acids for different classes of double substitutions. The DAS metric measures the difference in amino acid similarity/distance for the original➔final vs. original➔intermediate codons. DAS = AA similarity/distance (original➔final) − average AA similarity/distance (original➔intermediate). Three comparisons, using different amino acid similarity/distance matrices, are shown. **a** NN double substitutions. **b** SN double substitutions. **c** NS double substitutions

We hypothesized that the split of the NN into those evolving under positive selection, under purifying selection, or neutrally had to do with the (dis) similarity between the original, intermediate, and final amino acid residues (Fig. 3a). To test this hypothesis, we compared the differences in amino acid similarity/distance (DAS) between the subsets of NN, SN, and NS doubles for which positive selection, purifying selection, or a neutral evolution regime were detected (Fig. 5). This difference was calculated as DAS=*S*_of_ − *S*_oi_ where *S*_of_ and *S*_oi_ are the similarity/distance measures between the original and the final or intermediate amino acids, respectively. The measures of similarity between amino acid residues were extracted from 94 amino acid similarity/distance matrices that are available in the AAindex database [40]. In this database, some matrices are similarity matrices whereas others are distance matrices. For 85 of the 94 matrices, there was a significant difference between the DAS values of NN combinations under positive selection compared to those under purifying selection. For most of the double substitution combinations in the positively selected subset, DAS > 0, i.e., the final amino acid is significantly more similar to the original than the intermediate amino acids (in similarity matrices). Conversely, for most of the double substitution combinations in the negatively selected subset of the NN doubles, DAS < 0, i.e., the second mutation decreases the similarity of the amino acid in the given position to the original one. The significant differences were in the expected direction, where the values are opposite for the distance matrices compared to similarity matrices but reflect the same logic: positive selection for DAS > 0 for similarity matrices and DAS < 0 for distance matrices, and negative selection for the opposite case. Significant differences between positive vs. neutral, and neutral vs. negative subsets were observed as well albeit with fewer matrices (74 and 62, respectively). Focusing on 5 similarity/distance matrices that are based solely on physicochemical properties and thus rule out potential circular reasoning, we observed a significant difference between the DAS values for the NN combinations under positive and purifying selection, and between the combinations under positive selection and neutral evolution in all 5 matrices. However, the difference between the neutral combinations and those under purifying selection was not significant in 4 out of the 5 matrices.

We performed analogous comparisons also for the SN and NS classes of double substitutions. Although in each of the SN and NS combinations, there is only one non-synonymous, with only two amino acids involved, the DAS values can be formally calculated by including the ancestral vs. intermediate and the ancestral-final amino acid self-comparisons for SN and NS, respectively. For the SN combinations, 78 of the 94 matrices yielded a significant difference between the negative and neutral groups, i.e., the final amino acid is less similar to the original one in the combinations of purifying selection compared to neutral combinations. For 56 matrices, there was a significant difference between the positive and negative groups, but only 5 matrices showed a significant difference between the positive and neutral groups. All these significant differences were in the expected direction, depending on whether a particular matrix consists of similarity or distance values. For the 5 similarity/distance matrices that are based solely on physicochemical properties, only the difference between the neutral and negative groups was significant. For the NS combinations, there was no significant difference between the 15 combinations under positive selection and the single neutral combination. The lack of statistical support in the latter comparisons is most likely due to the small number of positive combinations for the SN class and, conversely, the dominance of positive selection in the NS class.

### Additional controls for mutational biases

For the SN, NS, and NN doubles, similar results were obtained when transitions and transversions were analyzed separately (Additional file 1: Figure S2) and when double substitutions in non-coding regions were used as null models instead of the NM1 or NM2 models (Additional file 1: Figure S3). The only exceptions were the SS doubles which had a greater DF compared to non-coding double substitutions (Additional file 1: Figure S2). This finding is likely explained by the purifying selection that, on average, affects non-coding regions to a greater extent than synonymous codon positions [41].

### Contribution of simultaneous double mutations

We estimated the frequency of simultaneous double mutations by calculating the difference between the observed double frequency in the null models, and the product of single synonymous substitutions (see the “Methods” section). The estimated frequencies of the double mutations range from 0 to 0.11, with the means of 0.0015 and 0.02 for NM2 and NM1, respectively. These frequencies are not negligible as they account for a mean of 53% of the double substitution frequency in NM2 and for 66% in NM1 (Additional file 1: Figure S6A). Although the DF is not significantly different between NM2 and NM1, the estimated proportion of simultaneous mutations is significantly greater in NM1 compared to NM2 (Additional file 1: Figure S6A). The product of the single substitution frequencies in the controls is nonetheless strongly correlated with the double frequency (Pearson’s correlation coefficients *r* = 0.93 for NM2 and r = 0.76 for NM1). A significant correlation was also observed between the single and double frequencies in all four classes of double substitutions (Additional file 1: Figure S7). We further verified that for the NS combinations, adjusting the DF by subtracting the estimated number of simultaneous double mutations (DM) from the observed double mutation count still resulted in significantly greater values compared to the null model (paired *t* test *p* value = 6.9 × 10^−04^ and signed rank test *p* value = 0.0011). This result (Additional file 1: Figure S8) presents further evidence that although simultaneous double mutations contribute to the observed double substitution frequency and to the DF, they cannot account for the elevated DF values in NS. Thus, the increase in DF in these combinations can only be attributed to positive selection.

## Discussion

The central goal of this work was to comprehensively characterize the selective landscape of codon double substitutions by accurately taking account of the mutational biases in the inference of selection. The control for mutation biases was achieved by comparing the DF for codon double substitutions to those of double synonymous substitutions. Previously analyzed codon double substitutions in serine codons [17] and in stop codons [16] suggest that these changes are under positive selection due to elevated double substitution frequencies compared to the expectation from single substitutions. Our focus here was to infer the type of selection by using more adequate controls, namely equivalent synonymous double substitutions, in order to address the possibility that apparent selection affecting codon double substitutions was due to mutational biases as previously suggested [9, 14]. Indeed, we observed that adjacent double synonymous substitutions (NM1) had a higher DF compared to the corresponding non-adjacent substitutions (NM2), although this difference was not statistically significant (Fig. 2e). We further estimated the double mutation rates for NM1 and NM2, and found that unlike the DF values, the double mutation rates were significantly different between the two null models (Additional file 1: Figure S6A). We estimated that simultaneous double mutations could account for ~ 70% of the double substitutions in the case of NM1 (adjacent synonymous) and ~ 40% for NM2 (non-adjacent synonymous) (Additional file 1: Figure S6B). The substantial rates of simultaneous double mutations indicate that the use of adequate null models that, in particular, explicitly take into account adjacency (as in NM1) is essential for estimating the effects of selection on double substitutions within codons. The obtained results are compatible with the previous estimates of multi-nucleotide mutation rates in eukaryotes [9]. However, it is likely that selection, on average, has a weaker effect on double substitutions in eukaryotes compared to prokaryotes due to the (typically) smaller effective population size of the former [42].

Partitioning of the codon double substitutions into four classes based on the (non) synonymy of the intermediate and final codon to the ancestral codon (SS, SN, NS, and NN) predicts the type of selection affecting the second step of the respective double substitutions (Fig. 3). In fact, this classification is a simple derivative of the classification of single substitutions in protein-coding genes into synonymous substitutions that are generally assumed to evolve neutrally, and non-synonymous substitutions most of which are subject to purifying selection [20] (Additional file 1: Figure S1). The classes of double substitutions are the four possible combinations of synonymous and non-synonymous substitutions at each step. Because the state resulting from the second step is the one for which selection is measured for by the DF comparison, the nature of this step largely defines the selective regime of the double substitution (Fig. 3a). Thus, SS doubles are effectively neutral. The SN doubles that drive an amino acid site away from the original state are generally subject to purifying selection, the strength of which depends on the similarity between the new amino acid introduced by the second substitution and the original amino acid. The few SN combinations that appear to be driven by positive selection all involve conservative amino acid replacements and might reflect a hitherto unrecognized process of adaptive fine-tuning of protein structures. Some NN substitutions could contribute to this process as well. Alternatively, this apparent positive selection could be an artifact caused by context-specific mutational biases. The NS doubles that return the site to the ancestral state are positively selected because, by definition, in all these combinations, the similarity of the final (same as ancestral) amino acid to the ancestral one is always greater compared to the intermediate. The NN doubles are heterogeneous, evolving either under purifying selection or under positive selection depending on which amino acid, intermediate or final, is more similar to the ancestral one. Notably, the DAS values are not always positive for the NN combinations under positive selection, as generally expected. This is most likely due to the fact that each amino acid substitution matrix accurately reflects similarity in certain properties but not others and thus does not equally well apply to all amino acid replacements. No single matrix is expected to be fully compatible with selection regimes on codon substitutions because they represent a mixture of numerous proteins from many environments that are subject to different sets of functional constraints.

Overall, the results of the present, comprehensive analysis of the evolutionary regimes of double substitutions reaffirm the predominantly conservative character of protein evolution [5, 43]. In bulk, all classes of double substitutions can be viewed as evolving under purifying selection if the double substitution is viewed as one evolutionary event. The positive selection detected for the second steps of the NS and many NN doubles is a consequence of the deleterious effect of the first substitution. The conclusion on the overall dominance of purifying selection is further supported by the comparison of double substitutions in fast vs. slow evolving genes. In accord with the identified purifying selection on SN combinations, these have significantly greater DF in fast evolving genes, similar to the higher rate of single non-synonymous changes in fast evolving genes compared to slow evolving ones. Conversely, those NS and NN substitutions, for which the second step was found to be driven by positive selection, showed a higher DF in slow evolving genes.

While alternative explanations of significantly greater DF in a codon double substitution compared to the control are plausible, our validation of positive selection in the NS class addresses them, including the possibility of context dependency to explain higher DF values and the possibility that the DF is higher because of unequal intermediate rates.

Compensation for the effects of deleterious mutations through subsequent positive selection has been previously hypothesized and demonstrated in other evolutionary contexts [44–46]. A major implication of the present results is that fitness valleys are commonly crossed in codon evolution as a result of positive selection that follows a deleterious non-synonymous mutation and that this route of evolution is, in large part, determined by the organization of the genetic code itself.

## Conclusion

Codon double substitutions are subject to distinct selection patterns depending on the combination of their (non) synonymy to the ancestral codon. The majority of combinations are under purifying selection because in most combinations the second substitution drives the codon to be more dissimilar than the first one. However, in combinations where the second substitution recovers a codon more similar to the ancestral codon, positive selection is observed. These results provide a framework for understanding the nature of double substitutions in codons, which were thus far only treated collectively as either supporting positive selection, or the consequence of multi-nucleotide mutations and thus only neutral. This new framework provides the basis to correctly infer selection in combinations on multi-nucleotide substitutions in protein-coding genes.

## Methods

### Datasets

Genomic data for bacteria and archaea were obtained from an updated version of the ATGC (Alignable Tight Genome Clusters) database [27]. For each genome cluster (ATGC), genes are grouped into clusters of orthologs, denoted by ATGC-COGs. Alignments of all sequences in each ATGC-COG (Cluster of Orthologous Genes) were constructed using the MAFFT v7.307 alignment software with the –linsi parameter [47]. To reconstruct the history of nucleotide substitutions in protein-coding DNA under the parsimony principle, we used triplets of closely related species [16, 17, 48]. Each triplet of species consists of two closely related species and an outgroup. Phylogenetic trees of all species triplets used in this study are shown in Additional file 1: Table S3. Distances between the two ingroup species are considerably smaller than the distances of the two ingroup species and the outgroup, in terms of the synonymous substations as measured by dS (see Additional file 1: Table S3). Only genes with exactly one ortholog per genome (1:1:1 orthologs) were included in all analyses. The genes were divided into slow and fast evolving ones by comparing the dN/dS value of each gene to the median dN/dS among all genome triplets in the given ATGC.

### Reconstruction of ancestral states using parsimony, and inferring the direction of change

From all triplet constructions, only triplets where the outgroup state is identical to at least one of the closely related species were considered. This configuration allows inference of the ancestral state of the two closely related species, using the parsimony principle (see Fig. 1). For example, the count of change AAA➔AGA was the number of combinations where one of the ingroup species had AGA whereas the other two species (including the outgroup) had AAA. The dataset included a total of 22,937,834 codons of which 3,338,517 (14.5%) had 1 substitution, 208,714 (0.9%) had 2 substitutions, and 49,926 (0.2%) had 3 substitutions based on the ancestral reconstruction.

In order to verify that ancestral reconstruction under the parsimony principle is adequate for the analysis of genomes within the range of distance characteristic of ATGCs, the subtree for each triplet was extracted from the full ATGC tree [27]. An artificial alignment was constructed such that an outgroup and an ingroup had identical sequences, whereas a second ingroup had a different sequence with either a single or double substitution per codon (Additional file 3). For each ATGC triplet, this alignment was used, in conjunction with the triplet phylogenetic tree to estimate the ancestral state of each codon using maximum likelihood (ML). The FastML.v3.11 software [49] with the Yang model for codons and without branch length optimization was employed to reconstruct codon evolution. For all triplets, the marginal probabilities of all codon reconstructions were 1, for the ancestral codon reconstructed with parsimony, thus validating the parsimony reconstructions.

### Analysis of codon double substitutions

The measure which is used to quantify the selection on double substitutions is the double fraction (DF), which is calculated for a specific double substitution, for example, AAA➔ GGA. Under the assumption that substitutions occur one at a time, observed double substitutions can result from two different paths (i.e., AAA➔ GAA➔ GGA or AAA➔ AGA➔ GGA in this example), as illustrated in Fig. 1A. The double fraction is the ratio between the number of instances of double substitutions and the sum of all instances, single and double substitutions. Specifically, the count of instances of a specific double substitution event (AAA➔ GGA) is noted by *b*. The counts of the respective single substitution intermediate states (AAA➔ GAA and AAA➔ AGA) are noted by *a*_1_ and *a*_2_. The double fraction is then:

$$ \mathrm{DF}=\frac{b}{a_1+{a}_2+b} $$

All states, intermediates and final, can be synonymous or non-synonymous with respect to the ancestral state, as described in the following subsection. The selection is inferred by comparing each DF to its corresponding null model, as explained below.

### Assignment of codon double substitution types

For each codon double substitution, there are two distinct paths from the ancestral codon state to the final (derived) codon state (Fig. 1A), where each step in the path represents a single substitution. The final and intermediate states can be either synonymous or non-synonymous, with respect to the ancestral state. Double substitutions where one of the intermediate states is a stop codon were excluded from the analysis. For example, the single substitution TCA➔ TAA is an intermediate state of the double substitution TCA➔ AAA, and therefore, the latter double substitution is not considered. All analyzed double substitutions were assigned to four classes, according the (non) synonymy of the intermediate and final states, compared to the inferred ancestral state. Specifically, the classes are noted by two letters, where the first letter indicates the (non) synonymy between the intermediate states and the ancestral, and the second letter indicates the relation between ancestral and final state. Each one of the letters can be N or S (standing for non-synonymous and synonymous, respectively), with a total of four possible combinations. All four combinations are illustrated in Fig. 3a. Accordingly, the four classifications are as follows:

1. SS—both intermediate single substitutions are synonymous, and the double substitution is also synonymous (Fig. 3a).
2. SN—at least one of the intermediate single substitutions is synonymous, where the other single substitution can be either synonymous or non-synonymous. The double substitution is non-synonymous to the ancestral state (Fig. 3a).
3. NS—one of the intermediate single substitutions is synonymous and the other non-synonymous. The double substitution is synonymous to the ancestral state (Fig. 3a).
4. NN—both intermediate single substitutions are non-synonymous, and the double substitution is non-synonymous to the ancestral state (Fig. 3a).

The special case where both intermediate single substitutions are non-synonymous, but the double substitution is synonymous to the ancestral state occurs only in some combinations of double substitutions where serine is the ancestral and final states. This special case was studied in details elsewhere [17]. For complementarity, the current analysis was also applied to the serine case (Additional file 1: Figure S2).

### Null model construction and analysis

Following the logic that single synonymous substitutions are the null model for non-synonymous substitutions, SS double substitutions combinations could in principle serve as the null model for other codon double substitutions. However, construction of more subtle null models is essential in the case of double substitutions, to address the following points. First, the number of instances of SS double substitutions in the dataset is limited (*n* = 8) to establish a reliable null model. In addition, construction of the null models described below allows more accurate comparisons between codon double substitutions and its corresponding null model in terms of base composition. Moreover, SS mutations are non-adjacent and occur only at positions 1 and 3 in the codon. Construction of null model where the two synonymous substitutions are adjacent allows more accurate comparison to the adjacent double substitutions in the codons, and accounts for the possibility of multi-nucleotide simultaneous mutations that occur in adjacent positions. Finally, synonymous double substitutions in a codon are co-dependent and therefore might be biased.

The first codon in NM1 (Fig. 2b, c) configurations can be any of the fourfold degenerate codons, i.e., codons for L, V, S, P, Y, A, R, and G, and the second codon in these configurations can be a codon for either R or L which are the only two amino acids that have a degenerate first codon position. Although in both NM1 configurations (Fig. 2b, c) the changes are in the same positions, the difference between the configuration in Fig. 2c and in Fig. 2c is the additional unchanged position which results in distinct context dependencies in the individual comparisons. An additional restriction for NM1 configurations is that the ancestral state of the third codon position of the second codon is a purine (A/G) because only then can the first codon substitution be synonymous. The first and second codons of the NM2 configuration (Fig. 2d) can be any of the fourfold degenerate codons.

Only codon double substitutions, for which a null model with the same nucleotide substitutions and nucleotide context (i.e., unchanging position) could be constructed, were included in the main analysis (e.g., serine codons were excluded because NM1 with adjacent synonymous substitutions with the same base composition as in serine is unavailable).

### Comparison of DF between codons and null models

It is critical to establish that the comparison of DF between codon double substitution and the null models produces results that are due to selection on the double substitutions (i.e., increase or decrease in the fixation rate of the second substitution) as opposed to negative selection removing single substitutions at such sites. The DF is estimated separately for codon double substitutions (DF_*n*_) and null model double substitutions (DF_*s*_) as follows:

$$ {\mathrm{DF}}_n=\frac{b_n}{a_{1n}+{a}_{2n}+{b}_n}=\frac{f\left({b}_n\right)}{f\left({a}_{1n}+{a}_{2n}+{b}_n\right)} $$

where $f\left({b}_n\right)=\frac{b_n}{\mathrm{ANC}n}$ and $f\left({a}_{1n}+{a}_{2n}+{b}_n\right)=\frac{a_{1n}+{a}_{2n}+{b}_n}{\mathrm{ANC}n}$ and ANC stands for the total count of the ancestral state of a specific codon, thus is used to calculate the frequency of the double and single substitutions denoted by *f*():

$$ {\mathrm{DF}}_s=\frac{b_s}{a_{1s}+{a}_{2s}+{b}_s}=\frac{f\left({b}_s\right)}{f\left({a}_{1s}+{a}_{2s}+{b}_s\right)} $$

In the extreme case when one of the intermediate states is strongly deleterious and thus never occurs, whereas the other intermediate and the final states are neutral (*f*(*a*_1*n*_) = *f*(*a*_1*s*_), *f*(*a*_2*n*_) = 0), the expected codon double frequency *f*(*b*_*n*_) is equivalent to half of the product of the null model double frequency *f*(*b*_*s*_) *f*(*b*_*n*_) *= 0.5f*(*b*_s_). Therefore, under neutrality:

$$ {\mathrm{DF}}_n=\frac{\frac{b_s}{2}}{a_{1s}+\frac{b_s}{2.}} $$

Thus, DF_*n*_ > DF_*s*_ when the N substitution (*a2n*) in the NS combination corresponds to the substitution with the relative higher rate in the null model (*a*_1*s*_ *< a*_2*s*_). This is the case for only 5 of the 16 NS combinations; in these 5 combinations, the greater DF compared to the control could result from negative selection on the intermediate.

Under neutrality, the observed double substitution frequency *f*(*b*) is expected to depend linearly on the single substitution rate (Additional file 1: Figure S7A-B), and thus, it is always expected to be higher in the null models than in codons. The DF, on the other hand, is the ratio of *b* and the sum of single and double substitutions, which provides an estimate of the rate of the substitutions from the intermediate to the final state. The correlation of DF to the sum of single and double substitutions is much weaker and is mostly observed in NM2 and SS (Additional file 1: Figure S7C). Although the observed frequency of double substitutions itself is not expected to exceed that of the null models, this is not the case for DF. Because DF accounts for the single substitutions through dividing the observed double substitution count by the sum of the double and single substitution counts (*a*_1_ + *a*_2_ + *b* is a correction for the single substitution rate—Additional file 1: Figure S4F), it can be and is indeed observed to exceed the value under null models when the second substitution in a codon double substitution combination exceeds the frequency of the second substitution in the null model. This excess is interpreted as an indication of positive selection, whereas a deficiency compared to the null model is interpreted as a sign of negative selection.

### Analysis of double substitutions in non-coding intergenic regions

While the main null models (NM1 and NM2) represented double synonymous substitutions from coding regions, we additionally compared the codon double substitutions to double substitutions in non-coding intergenic regions. The same analysis was performed in all possible frames of the aligned non-coding sequences as for the coding genes, treating triplets of bases as codons.

### Estimation of simultaneous double mutation frequency

In the null model configurations NM1 and NM2, the expected frequency of double substitutions, in the absence of simultaneous double mutations, can be estimated by the product of the frequencies of single synonymous substitutions. Because both single substitutions are synonymous, the effect of their order is assumed to be minimal. Thus, the estimated contribution of simultaneous double mutations in these combinations is the difference between the observed double substitution frequency and the product of the corresponding single substitutions (Additional file 1: Figure S8A). For 12 of the 634 combinations, the observed double frequency was smaller than the product of the corresponding single substitution frequencies; this is most likely due to noise, and accordingly, these combinations were ignored.

### Validation of selection in NS double substitutions

We further estimated an adjusted DF for the NS class of double substitutions by subtracting the estimated double mutation count (DM) from the observed count (*b*) and comparing the adjusted DF to the NM2 null model with the same base composition changes (Additional file 1: Figure S8). This adjusted DF analysis can only be performed for the NS and the SS class because only in these cases the ancestral and final codons are synonymous, and therefore, a multi-nucleotide mutation that would change the two bases simultaneously would be synonymous and thus expected to be (nearly) neutral. Conversely, multi-nucleotide mutations in the NN and SN combinations will be non-synonymous compared to the ancestral state and therefore are expected to be subject to negative selection. Accordingly, these combinations cannot be analyzed using DM which is estimated for synonymous substitutions that are assumed to be under no or minimal selection.

### Statistical tests

Two samples *t* test and the non-parametric Wilcoxon rank-sum test (*U* test) were used to compare the DF values between each of the codon double substitution types (SS, SN, NS, NN) and each of the null models (NM1, NM2) and between each of the codon double substitution types and adjacent and non-adjacent double substitutions in non-coding intergenic regions. Alpha level for significance was 0.01.

Paired *t* test and signed rank test were used to compare between the DF of different codon double substitution types in fast vs. slow evolving genes. Alpha level for significance was 0.01. Fisher’s exact test was used to compare the number of double codon substitutions to the sum of single and double substitutions, to test for significant differences in the DF between a specific codon double substitution and the comparable null model with the same base composition. For example, the codon double substitution GCC➔GTA, which changes the encoded amino acid from A to V, is compared to the null model of two adjacent synonymous substitutions (Fig. 2b) where the first base is G in the second codon position, followed by a synonymous C➔T change in a fourfold degenerate third codon position and by a synonymous C➔A change in the first codon position of the next codon (coding for R). The Benjamini–Hochberg procedure was used to correct for multiple testing, with alpha of 0.05.

## Supplementary information

**Additional file 1: Table S1.** Comparison of expected NS frequencies under neutrality to observed NM (null model) frequencies. **Table S2.** Individual comparison of NN codon double substitution. **Table S3.** Median distances (ds) between ingroup genomes and to the outgroup genome. **Figure S1.** Comparison of single synonymous (S) and non-synonymous (N) substitution frequencies. **Figure S2.** Comparison of codon double substitution classes to null models, separately by transitions and transversions. **Figure S3.** Comparison of codon double substitution classes to non-coding codon-like base triplets, separately by transitions and transversions. **Figure S4.** Double substitution compared to NM1 or NM2 for particular base combinations of double substitution. **Figure S5.** DF ratio compared to codon bias ratio in the SS class. **Figure S6.** Double mutation estimates compared between the null models. **Figure S7.** Observed double substitution frequencies and DF values compares to the DF denominator frequency. **Figure S8.** Adjusted DF comparison in the NS class after accounting for double mutations.

**Additional file 2.** Inference of the mode of selection for different types of double substitutions.

**Additional file 3.** Nucleotide sequence alignments constructed for the inference of ancestral states.

## Data Availability

All data generated or analyzed during this study are included in this published article and its supplementary information files. Genomic data for bacteria and archaea were obtained from an updated version of the ATGC (Alignable Tight Genome Clusters) database [27]; phylogenetic trees of all species triplets used in this study are shown in Additional file 1: Table S2.

## Abbreviations

BST: Branch-site test
DF: Double fraction
DAS: Differences in amino acid similarity
S: Synonymous
N: Non-synonymous
SS: Synonymous intermediate and synonymous final
NS: Non-synonymous intermediate and synonymous final
SN: Synonymous intermediate and non-synonymous final
NN: Non-synonymous intermediate and non-synonymous final
ATGC: Alignable Tight Genome Clusters
NM1: Synonymous substitution in the 3rd position of a codon followed by another synonymous substitution in the 1st position of the next codon
NM2: Non-adjacent synonymous substitutions in 3rd codon positions of consecutive codons
