# Supplementary material for: Crossing fitness valleys via double substitutions within codons
Source: BMC Biol. 2019 Dec 16;17:105. doi: 10.1186/s12915-019-0727-4 (PMC6916188; doi:10.1186/s12915-019-0727-4)
Supplement: Supplementary file 1 — Additional file 1: Table S1. Comparison of expected NS frequencies under neutrality to observed NM (null model) frequencies. Table S2. Individual comparison of NN codon double substitution. Table S3. Median distances (ds) between ingroup genomes and to the outgroup genome. Figure S1. Comparison of single synonymous (S) and non-synonymous (N) substitution frequencies. Figure S2. Comparison of codon double substitution classes to null models, separately by transitions and transversions. Figure S3. Comparison of codon double substitution classes to non-coding codon-like base triplets, separately by transitions and transversions. Figure S4. Double substitution compared to NM1 or NM2 for particular base combinations of double substitution. Figure S5. DF ratio compared to codon bias ratio in the SS class. Figure S6. Double mutation estimates compared between the null models. Figure S7. Observed double substitution frequencies and DF values compares to the DF denominator frequency. Figure S8. Adjusted DF comparison in the NS class after accounting for double mutations. [file 12915_2019_727_MOESM1_ESM.docx]

**Crossing fitness valleys via double substitutions within codons**

Frida Belinky^1^, Itamar Sela^1^, Igor B. Rogozin^1^, Eugene V. Koonin^1*^

^1^National Center for Biotechnology Information, National Library of Medicine, National Institutes of Health, Bethesda, Maryland, USA

^*^**To whom correspondence should be addressed. Email:** koonin@ncbi.nlm.nih.gov

Table S1 – Comparison of expected NS frequencies under neutrality to observed NM (null model) frequencies. Expected f(b_NS_) is calculated as follows:
 $expecte{d f(b}_{NS})=f\left( {a1}_{NM} \right)xf\left( {a2}_{NM} \right)x\frac{1}{2}+f\left( {a1}_{NS} \right)xf({a2}_{NM})x\frac{1}{2}$ where a1NS represents the nonsynonymous substitution count and f() represents the frequency which is calculated by division of a given count by the ancestral count (same as in figure S8). Similarly, a1NM represents the single synonymous frequency in the corresponding null model and a2NM represents the other single synonymous frequency in the corresponding null model. Thus the expected NS frequency is the sum of half the product of corresponding null model frequencies and the half of the product of nonsynonymous frequency and the counterpart synonymous frequency.

| ancestral codon | derived codon | f(a1NS) | f(a1NM) | f(a2NM) | expected f(bNS) | f(bNS) | f(bNM) | expected bNS | observed bNS | ANC NS | observed bNM | ANC NM | Fisher expected NS vs. observed NM | Fisher observed NS vs. observed NM |
| --- | --- | --- | --- | --- | --- | --- | --- | --- | --- | --- | --- | --- | --- | --- |
| CTC | TTA | 4.15E-03 | 6.35E-02 | 8.56E-03 | 2.89E-04 | 1.55E-03 | 1.73E-03 | 142 | 765 | 492000 | 61 | 35298 | 7.31E-24 | 0.4036 |
| CTT | TTA | 8.20E-03 | 1.28E-01 | 2.23E-02 | 1.52E-03 | 8.29E-03 | 6.38E-03 | 347 | 1889 | 228000 | 52 | 8147 | 4.64E-16 | 0.0695 |
| CTT | TTG | 8.20E-03 | 1.28E-01 | 2.10E-02 | 1.43E-03 | 7.25E-03 | 3.93E-03 | 326 | 1654 | 228000 | 32 | 8147 | 1.32E-06 | 0.0002 |
| CGC | AGA | 1.47E-03 | 7.22E-03 | 9.05E-03 | 3.93E-05 | 4.26E-04 | 2.01E-04 | 30 | 324 | 760000 | 131 | 650160 | 4.16E-20 | 5.98E-14 |
| CGC | AGG | 1.47E-03 | 7.22E-03 | 3.30E-02 | 1.43E-04 | 7.16E-04 | 4.52E-04 | 109 | 544 | 760000 | 294 | 650160 | 1.01E-27 | 1.21E-10 |
| AGA | CGT | 1.61E-03 | 2.78E-02 | 2.17E-02 | 3.19E-04 | 2.71E-03 | 1.57E-03 | 30 | 257 | 94700 | 85 | 54066 | 3.03E-16 | 6.22E-06 |
| AGA | CGC | 1.65E-03 | 2.12E-02 | 2.17E-02 | 2.48E-04 | 2.30E-03 | 1.15E-03 | 23 | 218 | 94700 | 62 | 54066 | 1.24E-11 | 3.87E-07 |
| CTC | TTG | 4.15E-03 | 6.35E-02 | 4.64E-02 | 1.57E-03 | 3.46E-03 | 2.92E-03 | 772 | 1702 | 492000 | 103 | 35298 | 4.58E-08 | 0.0985 |
| TTA | CTT | 3.22E-03 | 3.34E-02 | 4.68E-02 | 8.58E-04 | 6.09E-03 | 3.54E-03 | 232 | 1644 | 270000 | 78 | 22010 | 2.19E-21 | 5.42E-07 |
| CGT | AGA | 1.05E-03 | 1.69E-02 | 1.41E-02 | 1.26E-04 | 1.61E-03 | 1.03E-03 | 26 | 326 | 202000 | 79 | 76333 | 3.45E-24 | 0.0003 |
| TTA | CTC | 2.17E-03 | 2.02E-02 | 4.68E-02 | 5.24E-04 | 4.20E-03 | 2.09E-03 | 141 | 1135 | 270000 | 46 | 22010 | 6.65E-13 | 3.21E-07 |
| AGG | CGC | 2.64E-03 | 1.86E-02 | 3.09E-02 | 3.29E-04 | 5.98E-03 | 2.73E-03 | 12 | 220 | 36800 | 80 | 29349 | 2.60E-17 | 3.12E-10 |
| TTG | CTT | 2.81E-03 | 2.11E-02 | 1.14E-01 | 1.36E-03 | 5.84E-03 | 3.33E-03 | 277 | 1191 | 204000 | 54 | 16226 | 4.16E-08 | 1.57E-05 |
| CGT | AGG | 1.05E-03 | 1.41E-02 | 2.85E-02 | 2.16E-04 | 9.55E-04 | 1.10E-03 | 44 | 193 | 202000 | 84 | 76333 | 2.65E-19 | 0.2813675 |
| AGG | CGT | 2.47E-03 | 2.29E-02 | 3.09E-02 | 3.92E-04 | 3.10E-03 | 2.11E-03 | 14 | 114 | 36800 | 62 | 29349 | 5.16E-11 | 0.01500875 |
| TTG | CTC | 1.45E-03 | 1.70E-02 | 1.14E-01 | 1.05E-03 | 5.23E-03 | 5.18E-03 | 214 | 1066 | 204000 | 84 | 16226 | 3.76E-27 | 1 |

Table S2 – Individual comparison of NN codon double substitution to a single null model with the same mutational changes, and adjacency (NM1 for adjacent substitutions and NM2 for non-adjacent substitutions) using Fisher’s exact test

| codon change | | Null model – NM1 or NM2 | | | Codon change | | | Fisher double vs. single | Amino acid change | | | | mode of selection  after BH |
| --- | --- | --- | --- | --- | --- | --- | --- | --- | --- | --- | --- | --- | --- |
| ancestral | derived | double | single | ancestral | double | single | ancestral |  | ancestral | derived | inter-1 | inter-2 |  |
| CAG | AAC | 46 | 5089 | 72658 | 226 | 4185 | 630066 | 3.99E-37 | Q | N | H | K | + |
| TTC | CTG | 89 | 1853 | 16845 | 528 | 3052 | 590362 | 5.02E-34 | F | L | L | L | + |
| TTT | CTG | 44 | 1759 | 25416 | 257 | 2983 | 386236 | 4.7E-17 | F | L | L | L | + |
| TTA | ATT | 58 | 1227 | 22010 | 330 | 2355 | 269701 | 3.35E-16 | L | I | F | I | + |
| CAG | GAT | 38 | 2868 | 72658 | 208 | 4460 | 630066 | 1.72E-15 | Q | D | H | E | + |
| CAC | GAA | 74 | 4556 | 119063 | 62 | 1003 | 267623 | 3.47E-13 | H | E | Q | D | + |
| TTC | ATG | 22 | 637 | 16845 | 192 | 1325 | 590362 | 3.8E-13 | F | M | L | I | + |
| CAC | AAG | 78 | 5948 | 119063 | 82 | 1940 | 267623 | 8.99E-13 | H | K | Q | N | + |
| TTG | ATT | 14 | 621 | 16226 | 196 | 1705 | 204259 | 9.38E-13 | L | I | F | M | + |
| AAG | GCG | 42 | 1149 | 20988 | 296 | 2788 | 534568 | 1.94E-12 | K | A | T | E | + |
| ATA | CTG | 14 | 892 | 10829 | 169 | 2425 | 146332 | 1.86E-10 | I | L | M | L | + |
| TGG | GGC | 115 | 3048 | 51165 | 46 | 358 | 350601 | 6.71E-10 | W | G | C | G | + |
| TTT | ATG | 20 | 951 | 25416 | 146 | 1894 | 386236 | 1.09E-09 | F | M | L | I | + |
| ATT | CTG | 5 | 463 | 11031 | 338 | 4306 | 474289 | 1.53E-09 | I | L | M | L | + |
| GAG | AAT | 35 | 2623 | 70692 | 227 | 6505 | 668983 | 7.95E-09 | E | N | D | K | + |
| CAG | AAT | 12 | 1059 | 72658 | 178 | 3801 | 630066 | 1.22E-08 | Q | N | H | K | + |
| ATA | GTG | 45 | 1176 | 10829 | 506 | 5959 | 146332 | 4.23E-08 | I | V | M | V | + |
| GAC | CAA | 49 | 4127 | 80232 | 130 | 4726 | 700855 | 2.01E-07 | D | Q | E | H | + |
| ATC | CTG | 37 | 530 | 7969 | 905 | 5754 | 752044 | 3.30E-07 | I | L | M | L | + |
| CCG | GAG | 32 | 1876 | 45403 | 165 | 3851 | 580630 | 3.50E-07 | P | E | Q | A | + |
| ACG | CAG | 16 | 378 | 9609 | 166 | 1193 | 340066 | 4.98E-07 | T | Q | K | P | + |
| AAC | CAG | 31 | 771 | 11330 | 201 | 2010 | 436160 | 5.95E-07 | N | Q | K | H | + |
| GCG | CAG | 125 | 2103 | 79948 | 443 | 4544 | 907397 | 0.00000103 | A | Q | E | P | + |
| TTC | GTG | 35 | 839 | 16845 | 141 | 1386 | 590362 | 0.00000124 | F | V | L | V | + |
| ATT | TTG | 17 | 592 | 11031 | 236 | 2748 | 474289 | 0.00000131 | I | L | M | F | + |
| ATC | GTG | 38 | 777 | 7969 | 1549 | 15330 | 752044 | 0.00000226 | I | V | M | V | + |
| TTG | ATC | 19 | 560 | 16226 | 136 | 1366 | 204259 | 0.00000239 | L | I | F | M | + |
| TTA | ATC | 29 | 907 | 22010 | 149 | 1889 | 269701 | 0.00000262 | L | I | F | I | + |
| TTA | GTT | 24 | 1085 | 22010 | 113 | 1919 | 269701 | 0.0000038 | L | V | F | V | + |
| AAG | GAT | 31 | 1195 | 12030 | 154 | 2512 | 534568 | 0.00000458 | K | D | N | E | + |
| CAG | ACG | 20 | 740 | 14713 | 252 | 3516 | 630066 | 0.00000498 | Q | T | P | K | + |
| TGG | CGC | 346 | 7482 | 51165 | 51 | 521 | 350601 | 0.00000852 | W | R | C | R | + |
| ATC | TTG | 9 | 349 | 7969 | 204 | 2178 | 752044 | 0.0000113 | I | L | M | F | + |
| TTT | ATA | 48 | 1277 | 25416 | 138 | 1788 | 386236 | 0.0000148 | F | I | L | I | + |
| CAC | AAA | 25 | 1717 | 119063 | 58 | 1487 | 267623 | 0.0000283 | H | K | Q | N | + |
| ATG | CTT | 11 | 568 | 9430 | 290 | 4864 | 642679 | 0.0000305 | M | L | I | L | + |
| ATT | GTG | 22 | 795 | 11031 | 744 | 11951 | 474289 | 0.0000446 | I | V | M | V | + |
| ATA | TTG | 30 | 949 | 10829 | 179 | 2793 | 146332 | 0.000198 | I | L | M | L | + |
| TTT | GTA | 30 | 1196 | 25416 | 92 | 1724 | 386236 | 0.000308 | F | V | L | V | + |
| TTG | GTT | 21 | 995 | 16226 | 70 | 1393 | 204259 | 0.000309 | L | V | F | V | + |
| CAA | CGC | 24 | 672 | 12911 | 184 | 2490 | 270512 | 0.000525 | Q | R | H | R | + |
| ATG | GTC | 46 | 1463 | 9430 | 229 | 4236 | 642679 | 0.000627 | M | V | I | V | + |
| ACT | GTT | 353 | 5747 | 27743 | 215 | 2569 | 117239 | 0.000638 | T | V | I | A | + |
| CAG | GCG | 45 | 700 | 14713 | 469 | 4362 | 630066 | 0.00105 | Q | A | P | E | + |
| ATG | GTT | 36 | 1414 | 9430 | 176 | 3885 | 642679 | 0.0014 | M | V | I | V | + |
| AAC | CAA | 17 | 764 | 11330 | 109 | 2228 | 436160 | 0.00156 | N | Q | K | H | + |
| TGG | GGT | 98 | 3191 | 51165 | 23 | 338 | 350601 | 0.00168 | W | G | C | G | + |
| AAT | CAG | 14 | 676 | 14264 | 150 | 3176 | 333464 | 0.00201 | N | Q | K | H | + |
| AGT | GGG | 154 | 5198 | 56365 | 61 | 1254 | 114762 | 0.00217 | S | G | R | G | + |
| ATG | TTA | 35 | 819 | 9430 | 409 | 5609 | 642679 | 0.00221 | M | L | I | L | + |
| TTT | CTA | 51 | 2064 | 25416 | 114 | 2742 | 386236 | 0.00226 | F | L | L | L | + |
| CAC | CGA | 19 | 2948 | 20901 | 41 | 2743 | 267623 | 0.00253 | H | R | Q | R | + |
| ATG | CTC | 24 | 620 | 9430 | 377 | 5249 | 642679 | 0.00279 | M | L | I | L | + |
| AAC | GAA | 37 | 1177 | 11330 | 199 | 3737 | 436160 | 0.0028 | N | E | K | D | + |
| CAG | GAC | 281 | 7107 | 72658 | 247 | 4835 | 630066 | 0.00433 | Q | D | H | E | + |
| TTA | GTC | 11 | 781 | 22010 | 54 | 1575 | 269701 | 0.00478 | L | V | F | V | + |
| AAG | GAC | 49 | 1309 | 12030 | 134 | 2231 | 534568 | 0.0057 | K | D | N | E | + |
| AGT | GGA | 200 | 5605 | 56365 | 70 | 1313 | 114762 | 0.0058 | S | G | R | G | + |
| GAC | GGA | 4 | 953 | 8102 | 116 | 7884 | 700855 | 0.00658 | D | G | E | G | + |
| TGG | AGT | 109 | 2410 | 51165 | 19 | 203 | 350601 | 0.00743 | W | S | C | R | + |
| AAT | GAA | 55 | 1413 | 14264 | 367 | 6453 | 333464 | 0.00878 | N | E | K | D | + |
| TGG | AGC | 75 | 2216 | 51165 | 15 | 196 | 350601 | 0.0101 | W | S | C | R | + |
| TGG | CGT | 215 | 7511 | 51165 | 25 | 498 | 350601 | 0.0145 | W | R | C | R | + |
| TTC | ATA | 14 | 604 | 16845 | 46 | 954 | 590362 | 0.0149 | F | I | L | I | + |
| AGA | GGC | 106 | 4750 | 54066 | 15 | 328 | 94676 | 0.015 | R | G | S | G | + |
| GAG | ACG | 21 | 827 | 8129 | 243 | 5614 | 668983 | 0.0179 | E | T | A | K | + |
| TTG | GTC | 25 | 933 | 16226 | 53 | 1097 | 204259 | 0.0199 | L | V | F | V | + |
| CAC | GAG | 389 | 9049 | 119063 | 84 | 1454 | 267623 | 0.0211 | H | E | Q | D | + |
| AAT | CAA | 32 | 912 | 14264 | 211 | 3878 | 333464 | 0.0227 | N | Q | K | H | + |
| TTT | GTG | 25 | 893 | 25416 | 84 | 1814 | 386236 | 0.0286 | F | V | L | V | + |
| AGG | GGC | 128 | 3383 | 29349 | 18 | 265 | 36831 | 0.0345 | R | G | S | G | + |
| TTC | TCA | 8 | 469 | 10933 | 30 | 756 | 590362 | 0.0401 | F | S | L | S | + |
| GTT | TCT | 94 | 5428 | 38436 | 48 | 1915 | 250979 | 0.0429 | V | S | A | F | = |
| AGC | CGA | 65 | 2201 | 34345 | 25 | 1379 | 344526 | 0.0475 | S | R | R | R | = |
| AGA | GGT | 170 | 5169 | 54066 | 18 | 327 | 94676 | 0.0596 | R | G | S | G | = |
| CCT | ATT | 150 | 5975 | 136206 | 21 | 528 | 122662 | 0.0648 | P | I | L | T | = |
| GAG | CCG | 13 | 359 | 8129 | 134 | 6398 | 668983 | 0.0651 | E | P | A | Q | = |
| ATG | CTA | 44 | 1043 | 9430 | 218 | 7065 | 642679 | 0.0752 | M | L | I | L | = |
| AGC | TGG | 65 | 1799 | 34345 | 15 | 697 | 344526 | 0.0757 | S | W | R | C | = |
| CAA | AAC | 23 | 487 | 7534 | 121 | 3915 | 270512 | 0.0795 | Q | N | H | K | = |
| AGC | GGA | 122 | 3719 | 34345 | 128 | 4881 | 344526 | 0.0922 | S | G | R | G | = |
| GAC | CAG | 301 | 6756 | 80232 | 274 | 7085 | 700855 | 0.0971 | D | Q | E | H | = |
| GAC | GTA | 2 | 179 | 8102 | 12 | 4280 | 700855 | 0.108 | D | V | E | V | = |
| AAC | GAG | 64 | 1197 | 11330 | 141 | 3369 | 436160 | 0.124 | N | E | K | D | = |
| GAG | CAT | 40 | 3090 | 70692 | 69 | 7240 | 668983 | 0.141 | E | H | D | Q | = |
| AGC | CGG | 70 | 1960 | 34345 | 62 | 1332 | 344526 | 0.148 | S | R | R | R | = |
| ATG | TTC | 14 | 395 | 9430 | 187 | 3412 | 642679 | 0.15 | M | F | I | L | = |
| AGT | CGG | 41 | 2503 | 56365 | 11 | 396 | 114762 | 0.151 | S | R | R | R | = |
| CAA | GAT | 14 | 373 | 7534 | 162 | 2863 | 270512 | 0.178 | Q | D | H | E | = |
| CAA | AAT | 21 | 463 | 7534 | 236 | 3728 | 270512 | 0.179 | Q | N | H | K | = |
| AAA | GAT | 74 | 1884 | 20660 | 273 | 5775 | 574412 | 0.18 | K | D | N | E | = |
| CCG | AAG | 36 | 987 | 45403 | 90 | 1874 | 580630 | 0.18 | P | K | Q | T | = |
| GAC | GCA | 9 | 276 | 8102 | 114 | 5656 | 700855 | 0.19 | D | A | E | A | = |
| CTT | GCT | 305 | 2963 | 30749 | 67 | 786 | 228202 | 0.202 | L | A | P | V | = |
| TTT | ACT | 166 | 3584 | 78483 | 41 | 1113 | 386236 | 0.21 | F | T | S | I | = |
| AAT | GAG | 36 | 1176 | 14264 | 219 | 5664 | 333464 | 0.235 | N | E | K | D | = |
| TGT | GGG | 174 | 4761 | 100899 | 8 | 152 | 60699 | 0.281 | C | G | W | G | = |
| TGC | CGG | 463 | 13010 | 84580 | 16 | 343 | 154212 | 0.303 | C | R | W | R | = |
| TCT | ATT | 90 | 2307 | 31431 | 44 | 1365 | 134939 | 0.318 | S | I | F | T | = |
| TTT | GCT | 233 | 3461 | 78483 | 66 | 1120 | 386236 | 0.367 | F | A | S | V | = |
| TGT | AGG | 164 | 5460 | 100899 | 3 | 187 | 60699 | 0.377 | C | R | W | S | = |
| CAA | CCC | 9 | 317 | 12911 | 32 | 1628 | 270512 | 0.391 | Q | P | H | P | = |
| TGT | CGG | 228 | 8621 | 100899 | 5 | 129 | 60699 | 0.401 | C | R | W | R | = |
| ATG | GTA | 78 | 1898 | 9430 | 290 | 6272 | 642679 | 0.411 | M | V | I | V | = |
| GAA | GGC | 9 | 254 | 4630 | 217 | 7695 | 747383 | 0.445 | E | G | D | G | = |
| TTC | GTA | 17 | 796 | 16845 | 29 | 1049 | 590362 | 0.453 | F | V | L | V | = |
| CAC | CCA | 5 | 616 | 20901 | 11 | 863 | 267623 | 0.455 | H | P | Q | P | = |
| AGG | TGC | 58 | 1390 | 29349 | 3 | 126 | 36831 | 0.476 | R | C | S | W | = |
| ACT | CTT | 109 | 2255 | 27743 | 44 | 1050 | 117239 | 0.477 | T | L | I | P | = |
| AAG | CCG | 18 | 590 | 20988 | 92 | 3640 | 534568 | 0.486 | K | P | T | Q | = |
| AAA | CAT | 29 | 1109 | 20660 | 139 | 6043 | 574412 | 0.519 | K | H | N | Q | = |
| ATG | TTT | 18 | 360 | 9430 | 185 | 3112 | 642679 | 0.554 | M | F | I | L | = |
| CAT | GAG | 22 | 466 | 8000 | 59 | 1447 | 215209 | 0.598 | H | E | Q | D | = |
| GAT | CAG | 14 | 461 | 8454 | 195 | 5310 | 558773 | 0.602 | D | Q | E | H | = |
| GAA | AAT | 66 | 1321 | 11884 | 503 | 10709 | 747383 | 0.631 | E | N | D | K | = |
| CAA | GAC | 13 | 394 | 7534 | 91 | 3094 | 270512 | 0.64 | Q | D | H | E | = |
| CAT | AAG | 13 | 448 | 8000 | 76 | 2192 | 215209 | 0.666 | H | K | Q | N | = |
| CCT | GTT | 397 | 10755 | 136206 | 27 | 679 | 122662 | 0.676 | P | V | L | A | = |
| CAT | AAA | 15 | 339 | 8000 | 103 | 1993 | 215209 | 0.687 | H | K | Q | N | = |
| AGG | GGT | 140 | 3520 | 29349 | 11 | 252 | 36831 | 0.739 | R | G | S | G | = |
| GAC | AAA | 36 | 2751 | 80232 | 112 | 7939 | 700855 | 0.776 | D | K | E | N | = |
| AAG | CAT | 16 | 580 | 12030 | 90 | 3504 | 534568 | 0.778 | K | H | N | Q | = |
| ACG | GAG | 27 | 1076 | 9609 | 171 | 6313 | 340066 | 0.838 | T | E | K | A | = |
| CAA | CTC | 8 | 404 | 12911 | 33 | 1414 | 270512 | 0.849 | Q | L | H | L | = |
| AAA | GAC | 42 | 1572 | 20660 | 134 | 5205 | 574412 | 0.856 | K | D | N | E | = |
| AGT | TGG | 132 | 3959 | 56365 | 9 | 307 | 114762 | 0.868 | S | W | R | C | = |
| CAT | GAA | 20 | 353 | 8000 | 74 | 1236 | 215209 | 0.899 | H | E | Q | D | = |
| TTC | CTA | 42 | 1781 | 16845 | 58 | 2357 | 590362 | 0.919 | F | L | L | L | = |
| GAT | CAA | 10 | 343 | 8454 | 157 | 4950 | 558773 | 1 | D | Q | E | H | = |
| CAC | CTA | 3 | 428 | 20901 | 6 | 844 | 267623 | 1 | H | L | Q | L | = |
| AGT | CGA | 67 | 2890 | 56365 | 10 | 445 | 114762 | 1 | S | R | R | R | = |
| TCT | GTT | 125 | 2874 | 31431 | 45 | 2031 | 134939 | 0.0000908 | S | V | F | A | - |
| GCT | ATT | 1833 | 32582 | 417377 | 186 | 4440 | 232600 | 0.000115 | A | I | V | T | - |
| ACT | TTT | 102 | 1989 | 27743 | 37 | 1453 | 117239 | 0.000212 | T | F | I | S | - |
| GAA | CAT | 19 | 522 | 11884 | 107 | 7891 | 747383 | 0.00034 | E | H | D | Q | - |
| CTT | ACT | 96 | 2792 | 30749 | 42 | 2305 | 228202 | 0.000502 | L | T | P | I | - |
| GAC | AAG | 114 | 5206 | 80232 | 142 | 10184 | 700855 | 0.000516 | D | K | E | N | - |
| TCT | CTT | 273 | 4820 | 31431 | 41 | 1274 | 134939 | 0.000557 | S | L | F | P | - |
| GCT | CTT | 952 | 23864 | 417377 | 55 | 2171 | 232600 | 0.000837 | A | L | V | P | - |
| AAG | CAC | 41 | 701 | 12030 | 101 | 3254 | 534568 | 0.00124 | K | H | N | Q | - |
| ATT | TCT | 90 | 2785 | 44754 | 30 | 1691 | 474289 | 0.00403 | I | S | T | F | - |
| GAG | AAC | 162 | 5662 | 70692 | 162 | 7829 | 668983 | 0.00437 | E | N | D | K | - |
| GAA | GTC | 6 | 167 | 4630 | 55 | 6177 | 747383 | 0.00578 | E | V | D | V | - |
| AGC | GGG | 135 | 3486 | 34345 | 133 | 4802 | 344526 | 0.00822 | S | G | R | G | - |
| GCT | TTT | 477 | 18909 | 417377 | 63 | 3540 | 232600 | 0.00836 | A | F | V | S | - |
| GAA | AAC | 51 | 1314 | 11884 | 297 | 11359 | 747383 | 0.0128 | E | N | D | K | - |
| TGC | AGG | 94 | 2993 | 84580 | 4 | 398 | 154212 | 0.0153 | C | R | W | S | - |
| GCG | AAG | 99 | 2973 | 79948 | 278 | 11089 | 907397 | 0.0183 | A | K | E | T | - |
| TGC | GGG | 255 | 4347 | 84580 | 8 | 307 | 154212 | 0.0192 | C | G | W | G | - |
| AAA | CAC | 22 | 822 | 20660 | 88 | 5561 | 574412 | 0.0321 | K | H | N | Q | - |
| ATT | GCT | 524 | 4640 | 44754 | 182 | 10538 | 474289 | 2.06E-119 | I | A | T | V | - |
| GAG | CAC | 342 | 6304 | 70692 | 102 | 8662 | 668983 | 3.23E-49 | E | H | D | Q | - |
| TTT | CCT | 336 | 5448 | 78483 | 33 | 2083 | 386236 | 2.92E-18 | F | P | S | L | - |
| ATT | CCT | 99 | 2502 | 44754 | 27 | 3144 | 474289 | 1.14E-14 | I | P | T | L | - |
| GAA | CAC | 36 | 547 | 11884 | 98 | 8738 | 747383 | 2.05E-14 | E | H | D | Q | - |
| GTT | ACT | 348 | 7731 | 38436 | 239 | 9770 | 250979 | 6.9E-13 | V | T | A | I | - |
| GTT | CCT | 199 | 5543 | 38436 | 18 | 2026 | 250979 | 2E-11 | V | P | A | L | - |
| CTT | TCT | 255 | 5036 | 30749 | 39 | 2045 | 228202 | 7.51E-10 | L | S | P | F | - |
| GAT | AAG | 46 | 982 | 8454 | 172 | 11659 | 558773 | 7.67E-10 | D | K | E | N | - |
| CCT | TTT | 656 | 10829 | 136206 | 38 | 1458 | 122662 | 3.03E-08 | P | F | L | S | - |
| GAT | AAA | 46 | 868 | 8454 | 277 | 11442 | 558773 | 0.00000943 | D | K | E | N | - |

Table S3 – Median distances (ds) between ingroup genomes (ds within) and the average of medians of each of the ingroup genomes to the outgroup genome (ds between) for each ATGC triplet. For each ATGC triplet an ML tree extracted from the full tree as originally reconstructed for the entire ATGC group.

|  | dS within | dS between | tree |
| --- | --- | --- | --- |
| ATGC001 | 0.207 | 0.407 | (Salmonella_bongori_NCTC_12419_uid70155:0.054991,(Salmonella_enterica_arizonae_serovar_62_z4_z23__uid58191:0.031031,Salmonella_enterica_serovar_Choleraesuis_SC_B67_uid58017:0.032358):0.022201); |
| ATGC003 | 0.187 | 0.262 | ((Streptococcus_pseudopneumoniae_IS7493_uid71153:0.027231,Streptococcus_pneumoniae_INV200_uid162035:0.042):0.00937580952380952,Streptococcus_mitis_B6_uid46097:0.0481551904761905); |
| ATGC008 | 0.112 | 0.467 | ((Streptococcus_salivarius_JIM8777_uid162145:0.012791,Streptococcus_salivarius_CCHSS3_uid70481:0.021592):0.0427026666666667,Streptococcus_thermophilus_CNRZ1066_uid58221:0.0607703333333333); |
| ATGC014 | 0.202 | 0.527 | (Bacillus_weihenstephanensis_KBAB4_uid58315:0.0816286379310345,(Bacillus_cereus_ATCC_10987_uid57673:0.037554,Bacillus_thuringiensis_Al_Hakam_uid58795:0.037617):0.0380243620689655); |
| ATGC015 | 0.141 | 0.258 | (Bacillus_subtilis_spizizenii_W23_uid51879:0.033412,(Bacillus_subtilis_BSP1_uid184010:0.025965,Bacillus_JS_uid162189:0.024521):0.020041); |
| ATGC035 | 0.307 | 0.543 | ((Mycoplasma_capricolum_ATCC_27343_uid58525:0.050541,Mycoplasma_leachii_PG50_uid60849:0.026182):0.0364871666666667,Mycoplasma_mycoides_SC_PG1_uid58031:0.0663658333333333); |
| ATGC044 | 0.242 | 0.594 | (Rickettsia_prowazekii_Rp22_uid161945:0.0870167564102564,(Rickettsia_akari_Hartford_uid58161:0.062083,Rickettsia_massiliae_MTU5_uid58801:0.032325):0.0476992435897436); |
| ATGC050 | 0.254 | 0.858 | (Helicobacter_cetorum_MIT_99_5656_uid162215:0.16663652,(Helicobacter_pylori_83_uid161153:0.112778,Helicobacter_pylori_SouthAfrica7_uid159989:0.028691):0.07033548); |
| ATGC071 | 0.282 | 0.53 | (Pseudomonas_putida_GB_1_uid58735:0.057752,(Pseudomonas_putida_H8234_uid208673:0.035811,Pseudomonas_putida_NBRC_14164_uid208670:0.029301):0.024501); |
| ATGC088 | 0.281 | 0.338 | ((Burkholderia_thailandensis_E264_uid58081:0.031471,Burkholderia_thailandensis_MSMB121_uid201037:0.029171):0.00682090909090909,Burkholderia_pseudomallei_K96243_uid57733:0.0368450909090909); |
| ATGC089 | 0.234 | 0.512 | (Burkholderia_cepacia_GG4_uid173858:0.046212,(Burkholderia_cenocepacia_J2315_uid57953:0.020621,Burkholderia_cenocepacia_MC0_3_uid58769:0.023482):0.041562); |
| ATGC097 | 0.264 | 0.596 | (Erwinia_tasmaniensis_Et1_99_uid59029:0.0824487,(Erwinia_amylovora_ATCC_49946_uid46943:0.051112,Erwinia_Ejp617_uid159955:0.041762):0.0372823); |
| ATGC100 | 0.337 | 0.441 | ((Pectobacterium_carotovorum_PC1_uid59295:0.052141,Pectobacterium_carotovorum_PCC21_uid174335:0.044871):0.0164033333333333,Pectobacterium_SCC3193_uid193707:0.0687296666666667); |
| ATGC104 | 0.119 | 1.01 | (Bifidobacterium_breve_ACS_071_V_Sch8b_uid158863:0.105430055555556,(Bifidobacterium_longum_infantis_ATCC_15697_uid159865:0.033943,Bifidobacterium_longum_JCM_1217_uid62695:0.020585):0.0834319444444444); |
| ATGC108 | 0.283 | 0.819 | ((Listeria_monocytogenes_SLCC2376_uid175111:0.031602,Listeria_monocytogenes_J0161_uid54459:0.064007):0.060841,Listeria_innocua_Clip11262_uid61567:0.077141); |
| ATGC111 | 0.137 | 0.806 | ((Aeromonas_hydrophila_ATCC_7966_uid58617:0.015081,Aeromonas_hydrophila_ML09_119_uid205540:0.014921):0.054461,Aeromonas_salmonicida_A449_uid58631:0.087611); |
| ATGC123 | 0.215 | 0.527 | (Rhizobium_leguminosarum_bv__trifolii_WSM2304_uid58997:0.054901,(Rhizobium_leguminosarum_bv__trifolii_WSM1325_uid58991:0.029291,Rhizobium_leguminosarum_bv__viciae_3841_uid57955:0.029351):0.039161); |
| ATGC125 | 0.332 | 0.533 | (Sinorhizobium_fredii_USDA_257_uid168059:0.0625885,(Rhizobium_NGR234_uid59081:0.047911,Sinorhizobium_fredii_HH103_uid86865:0.037141):0.0200625); |
| ATGC134 | 0.197 | 0.427 | (Xanthomonas_oryzae_oryzicola_BLS256_uid54411:0.056132,(Xanthomonas_axonopodis_citrumelo_F1_uid73179:0.027672,Xanthomonas_axonopodis_citri_306_uid57889:0.028283):0.029141); |
| ATGC135 | 0.274 | 0.499 | (Stenotrophomonas_maltophilia_R551_3_uid58657:0.064039,(Stenotrophomonas_maltophilia_D457_uid162199:0.034501,Stenotrophomonas_maltophilia_JV3_uid72473:0.030391):0.024983); |
| ATGC137 | 0.154 | 0.208 | ((Neisseria_lactamica_020_06_uid60851:0.053671,Neisseria_gonorrhoeae_TCDC_NG08107_uid161097:0.029383):0.00128680357142857,Neisseria_meningitidis_MC58_uid57817:0.0420721964285714); |
| ATGC138 | 0.319 | 0.698 | (Francisella_TX077308_uid68321:0.076341,(Francisella_noatunensis_orientalis_Toba_04_uid164779:0.051571,Francisella_philomiragia_ATCC_25017_uid59105:0.033751):0.042641); |
| ATGC144 | 0.247 | 0.388 | (Borrelia_garinii_BgVir_uid162165:0.0622729,(Borrelia_burgdorferi_ZS7_uid59429:0.038464,Borrelia_bissettii_DN127_uid71231:0.040961):0.0223211); |
| ATGC147 | 0.416 | 0.527 | ((Methanococcus_maripaludis_C5_uid58741:0.060151,Methanococcus_maripaludis_C6_uid58947:0.051021):0.017331,Methanococcus_maripaludis_C7_uid58847:0.061551); |
| ATGC149 | 0.436 | 0.805 | ((Acinetobacter_calcoaceticus_PHEA_2_uid83123:0.044711,Acinetobacter_oleivorans_DR1_uid50119:0.054731):0.030421375,Acinetobacter_baumannii_AB307_0294_uid59271:0.073842625); |
| ATGC165 | 0.116 | 1.106 | ((Rhodobacter_sphaeroides_KD131_uid59277:0.010061,Rhodobacter_sphaeroides_ATCC_17029_uid58449:0.015152):0.0812863333333333,Rhodobacter_sphaeroides_ATCC_17025_uid58451:0.0949646666666667); |
| ATGC171 | 0.184 | 0.384 | (Thermoanaerobacter_mathranii_A3_uid49481:0.0571863,(Thermoanaerobacter_X514_uid58589:0.032643,Thermoanaerobacter_wiegelii_Rt8_B1_uid52581:0.038421):0.0236657); |
| ATGC177 | 0.265 | 0.555 | (Prochlorococcus_marinus_MIT_9312_uid58357:0.069103,(Prochlorococcus_marinus_AS9601_uid58307:0.037711,Prochlorococcus_marinus_MIT_9301_uid58437:0.041811):0.028189); |
| ATGC181 | 0.249 | 0.434 | (Caldicellulosiruptor_owensensis_OL_uid60165:0.078382,(Caldicellulosiruptor_hydrothermalis_108_uid60157:0.043822,Caldicellulosiruptor_bescii_DSM_6725_uid59201:0.060262):0.018611); |
| ATGC188 | 0.34 | 0.404 | (Ralstonia_solanacearum_CFBP2957_uid50545:0.039112,(Ralstonia_solanacearum_GMI1000_uid57593:0.037491,Ralstonia_solanacearum_PSI07_uid50539:0.030281):0.012301); |
| ATGC189 | 0.174 | 0.681 | ((Ralstonia_eutropha_H16_uid62925:0.021091,Cupriavidus_necator_N_1_uid68689:0.024231):0.041765,Cupriavidus_taiwanensis_LMG_19424_uid61615:0.064426); |
| ATGC199 | 0.338 | 0.583 | (Paenibacillus_terrae_HPL_003_uid82371:0.0814463333333333,(Paenibacillus_polymyxa_SC2_uid59583:0.061372,Paenibacillus_polymyxa_E681_uid53477:0.042191):0.0264746666666667); |
| ATGC201 | 0.373 | 0.818 | (Bartonella_quintana_Toulouse_uid57635:0.088333,(Bartonella_grahamii_as4aup_uid59405:0.041991,Bartonella_tribocorum_CIP_105476_uid59129:0.058011):0.084522); |
| ATGC210 | 0.384 | 0.543 | ((Dehalococcoides_VS_uid42393:0.052801,Dehalococcoides_ethenogenes_195_uid57763:0.058941):0.0221214,Dehalococcoides_GT_uid42115:0.0780026); |
| ATGC213 | 0.159 | 0.755 | (Methylobacterium_populi_BJ001_uid58937:0.0725065,(Methylobacterium_extorquens_PA1_uid58821:0.018142,Methylobacterium_extorquens_AM1_uid57605:0.018402):0.0542645); |
| ATGC234 | 0.543 | 0.586 | (Clavibacter_michiganensis_sepedonicus_uid61577:0.0456785,(Clavibacter_michiganensis_NCPPB_382_uid61625:0.040681,Clavibacter_michiganensis_nebraskensis_NCPPB_2581_uid195908:0.044211):0.0032325); |
| ATGC252 | 0.136 | 0.381 | (Anaeromyxobacter_dehalogenans_2CP_C_uid58135:0.037991,(Anaeromyxobacter_K_uid58953:0.013591,Anaeromyxobacter_dehalogenans_2CP_1_uid58989:0.014331):0.02403); |


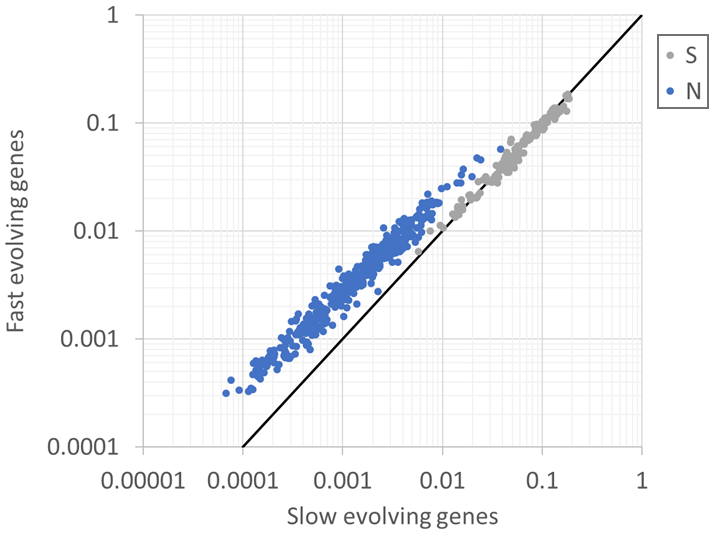


**Figure S1.** Comparison of single synonymous (S) and non-synonymous (N) substitution frequencies in fast evolving genes (with dN/dS>median) vs. frequencies in slow evolving genes (dN/dS<media).


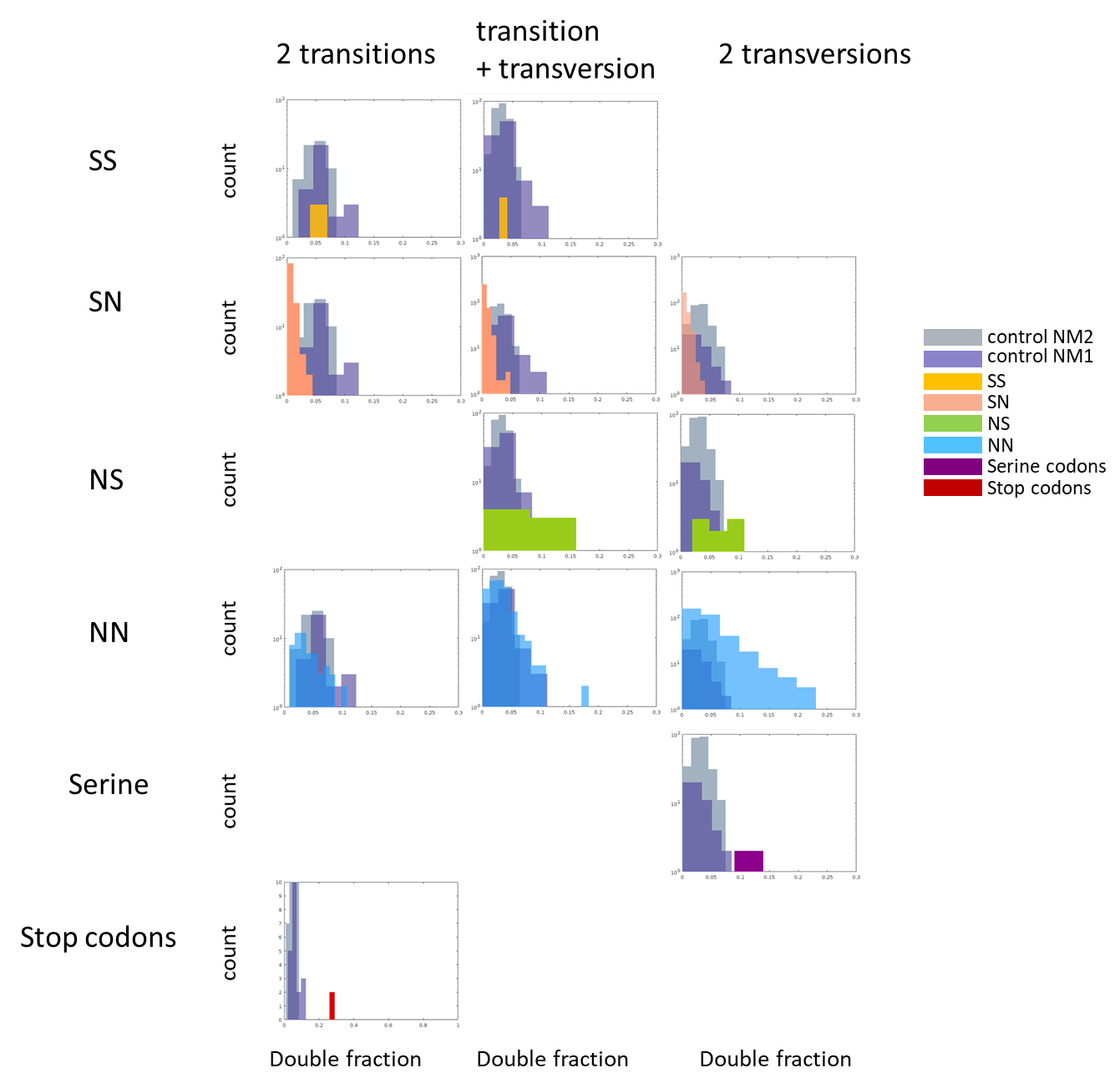


**Figure S2.** Comparison of each of the codon double substitution classes to the double synonymous null models, separately by transitions and transversions.


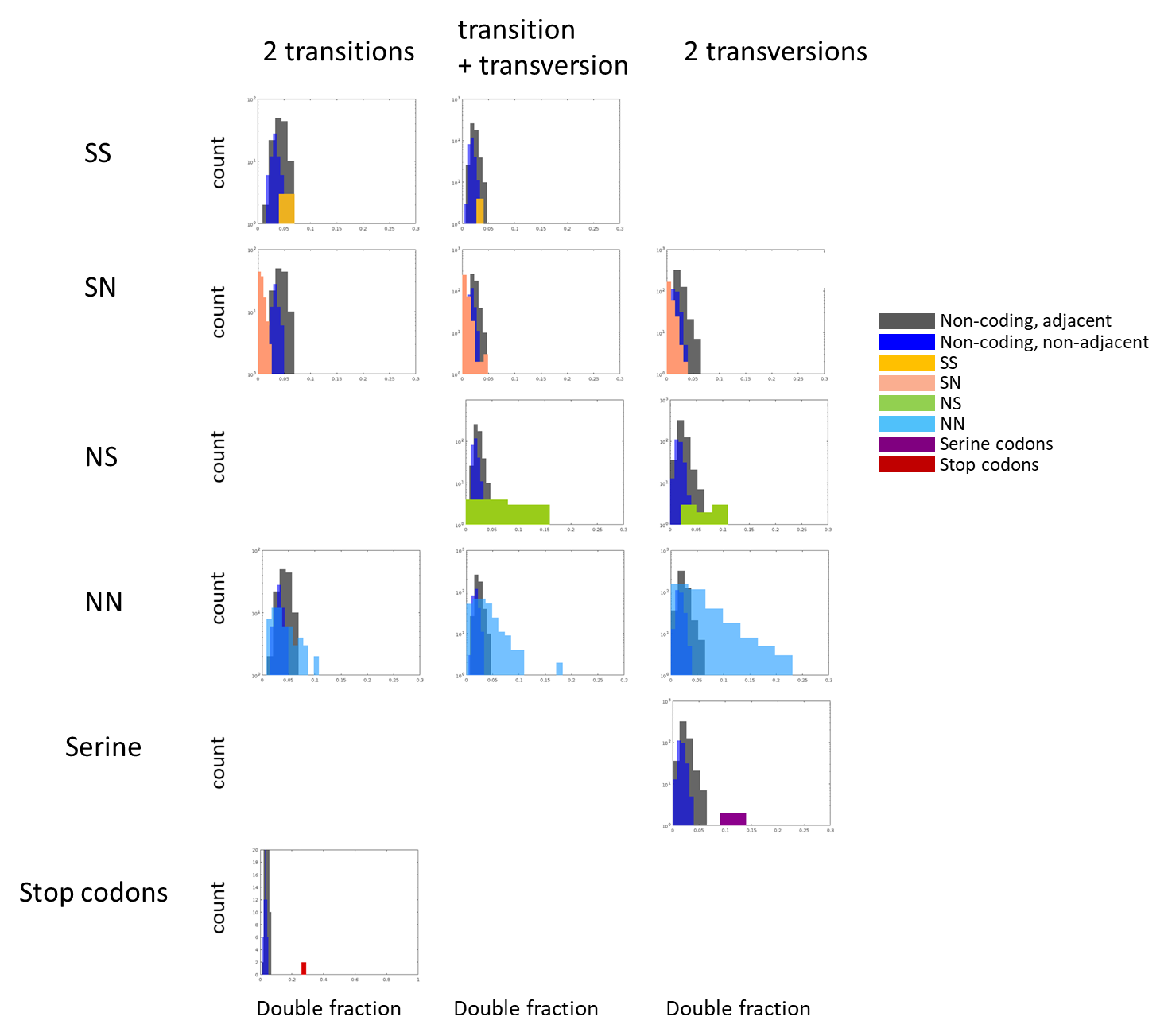


**Figure S3.** Comparison of each of the codon double substitution classes to non-coding codon-like base triplets, separately by transitions and transversions.

**
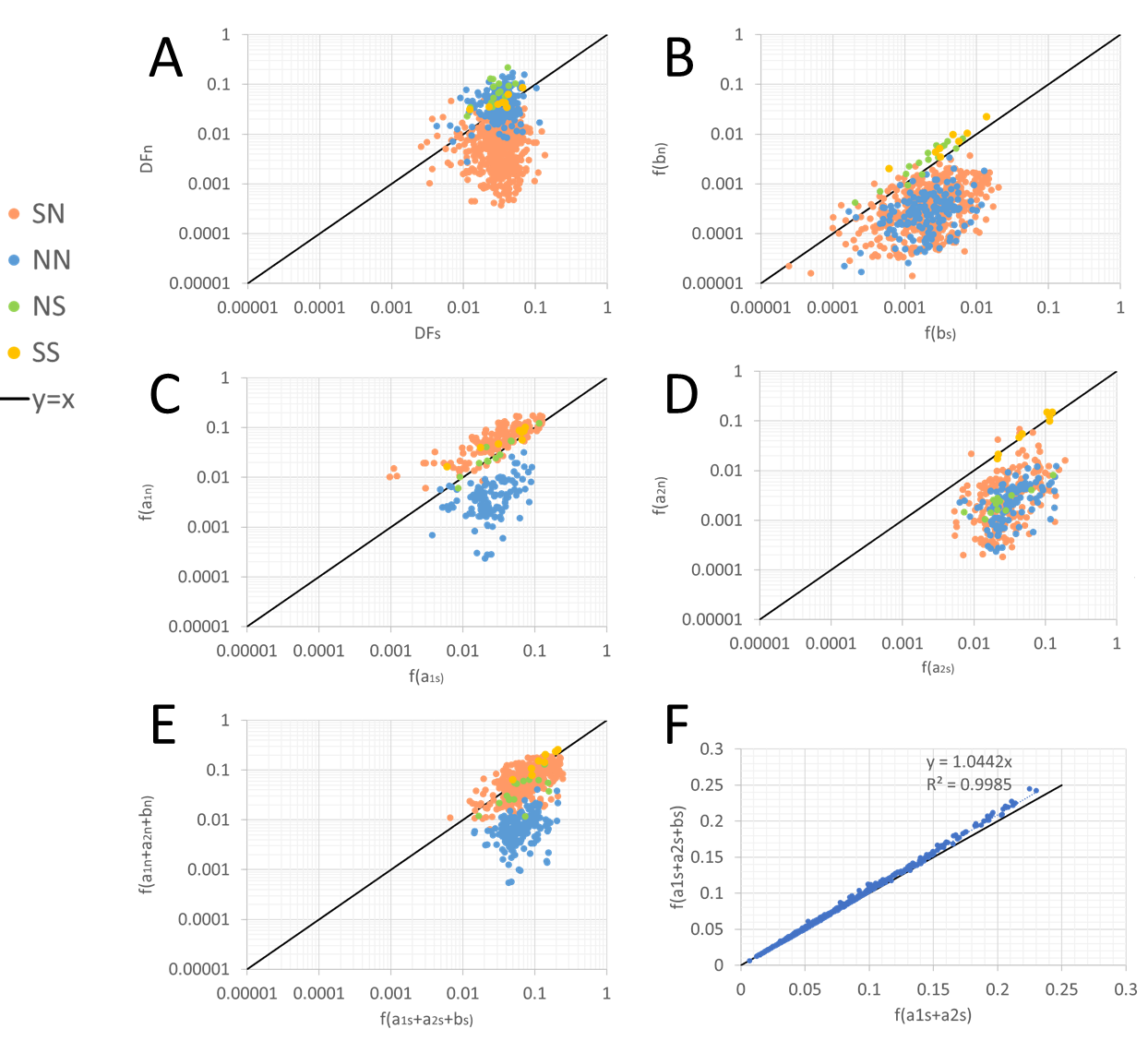
**
**Figure S4.** Each point represents a particular base combination of double substitution compared between this double change in a codon and this double change in NM1 or NM2. Points are colored by their classification to the four codon double substitution classes. (A) comparison of DFn (DF in codons) to DFs (DF in null models). (B) comparison of f(b) – the observed frequency of double substitutions in codons (bn) vs. that in the null models (bs). (C) comparison of a1 frequency – defined as the more frequent single substitution in codons (a1n) vs. that in the null models (a1s). (D) comparison of a2 frequency – defined as the less frequent single substitution in codons (a2n) vs. that in the null models (a2s). (E) comparison of the sum of double and single substitution per codon vs. the sum in the equivalent null model. (F) comparison of the sum of double and single substitutions in the null models to the sum of single substitutions in the null models.


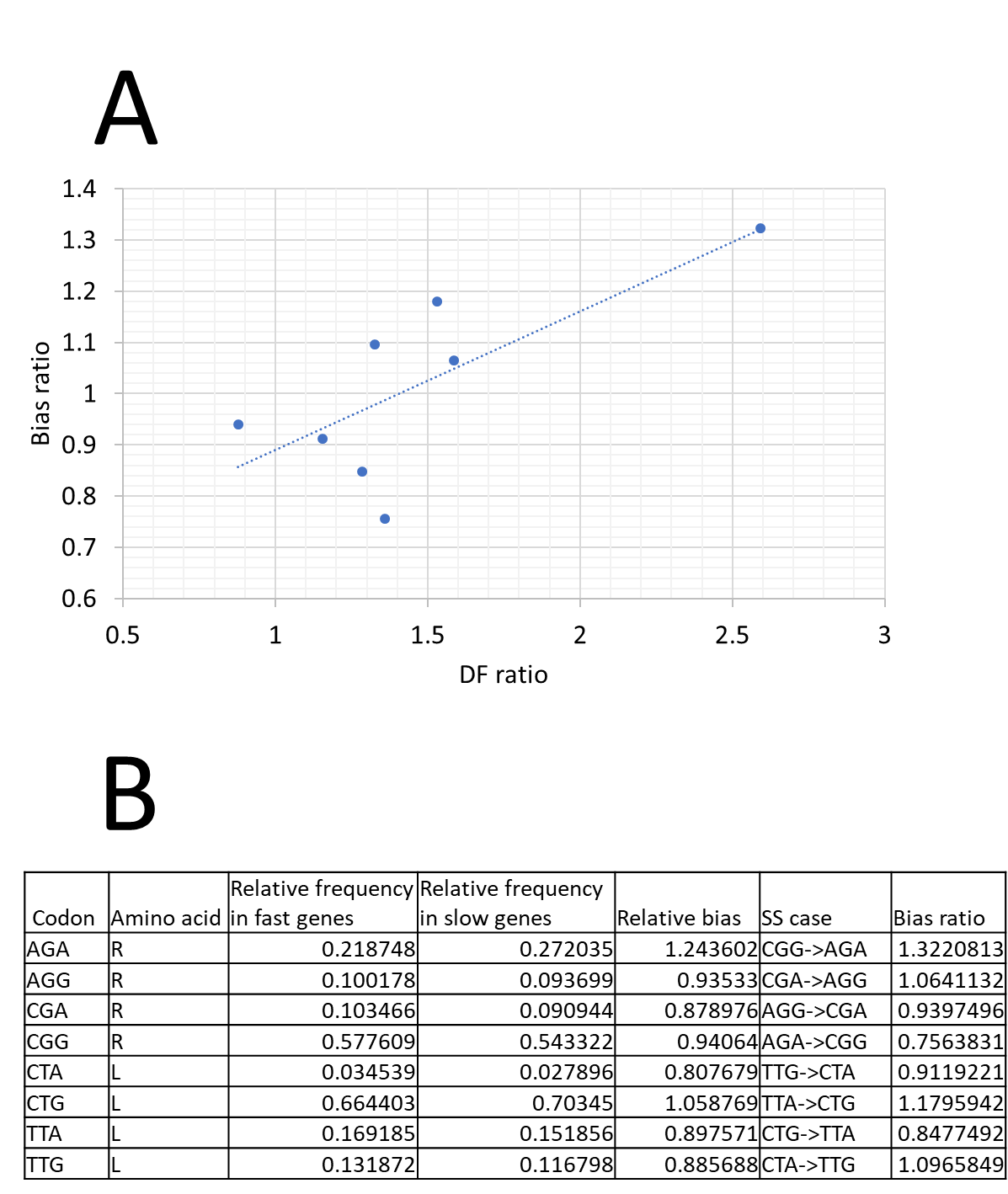


**Figure S5.** (A) Pearson correlation (R=0.74, p-val=0.037) between selection strength in the SS class, estimated by the DF ratio (ratio between the individual codon DFn and the control DFs- individual cases of NM2), and the codon bias ratio. (B) The bias ratio is calculated as the ratio between the biases of the final codon compared to the original one, based on relative biases of these codon calculated as the ratio of their relative frequency in slow vs. fast evolving genes (see methods for detailed explanation on fast and slow genes).


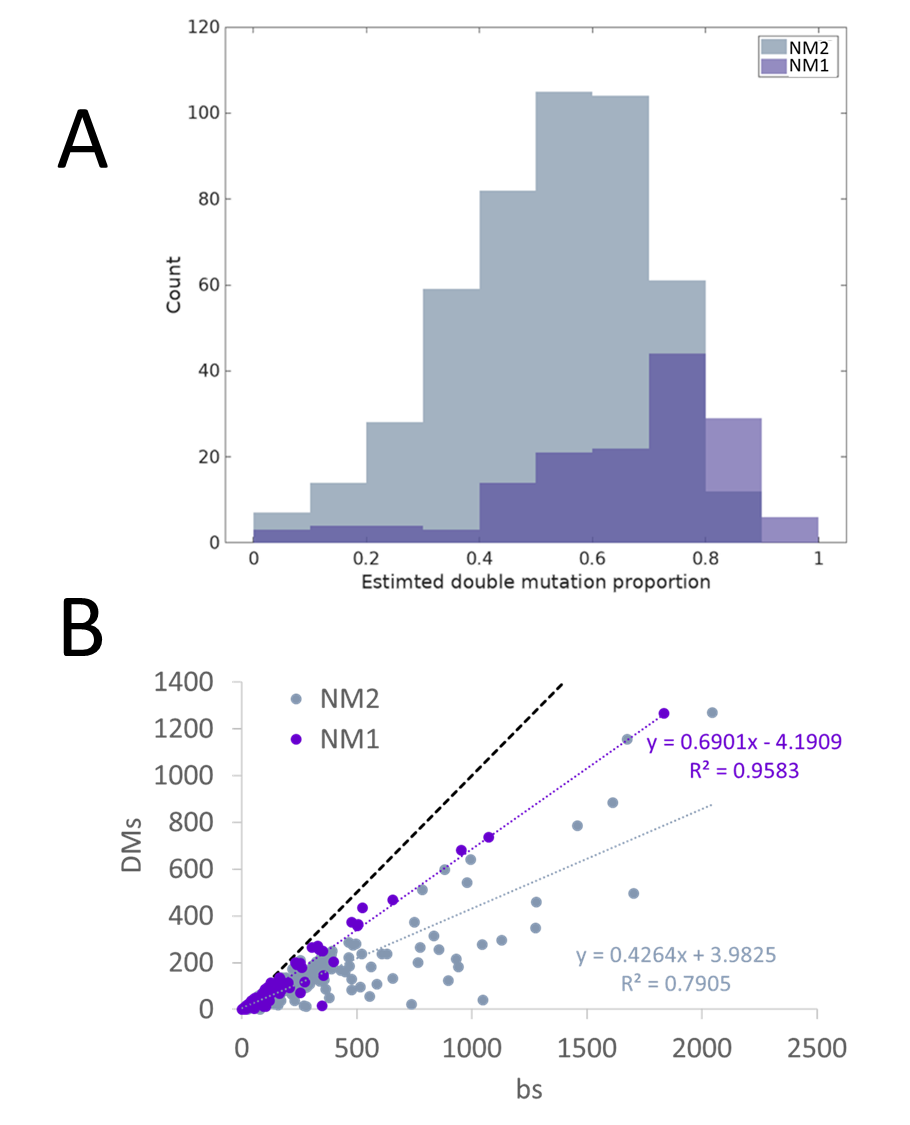


**Figure S6.** (A) Proportion of the estimated double mutation frequency out of the observed double substitution frequency in the null models NM1 (adjacent synonymous substitutions) and NM2 (non-adjacent synonymous substitutions). The difference between NM1 and NM2 distributions is significant (p-val= 9.98x10^-15^ for t-test and p-val= 2.02x10^-16^ for rank sum test). (B) DMs – Double mutation estimated counts in controls NM1 and NM2 compared to bs – double substitution counts in NM1 and NM2. Multi-nucleotide mutations are estimated to accounts for 70% of adjacent synonymous double substitutions and for 40% of close non-adjacent synonymous double substitutions.


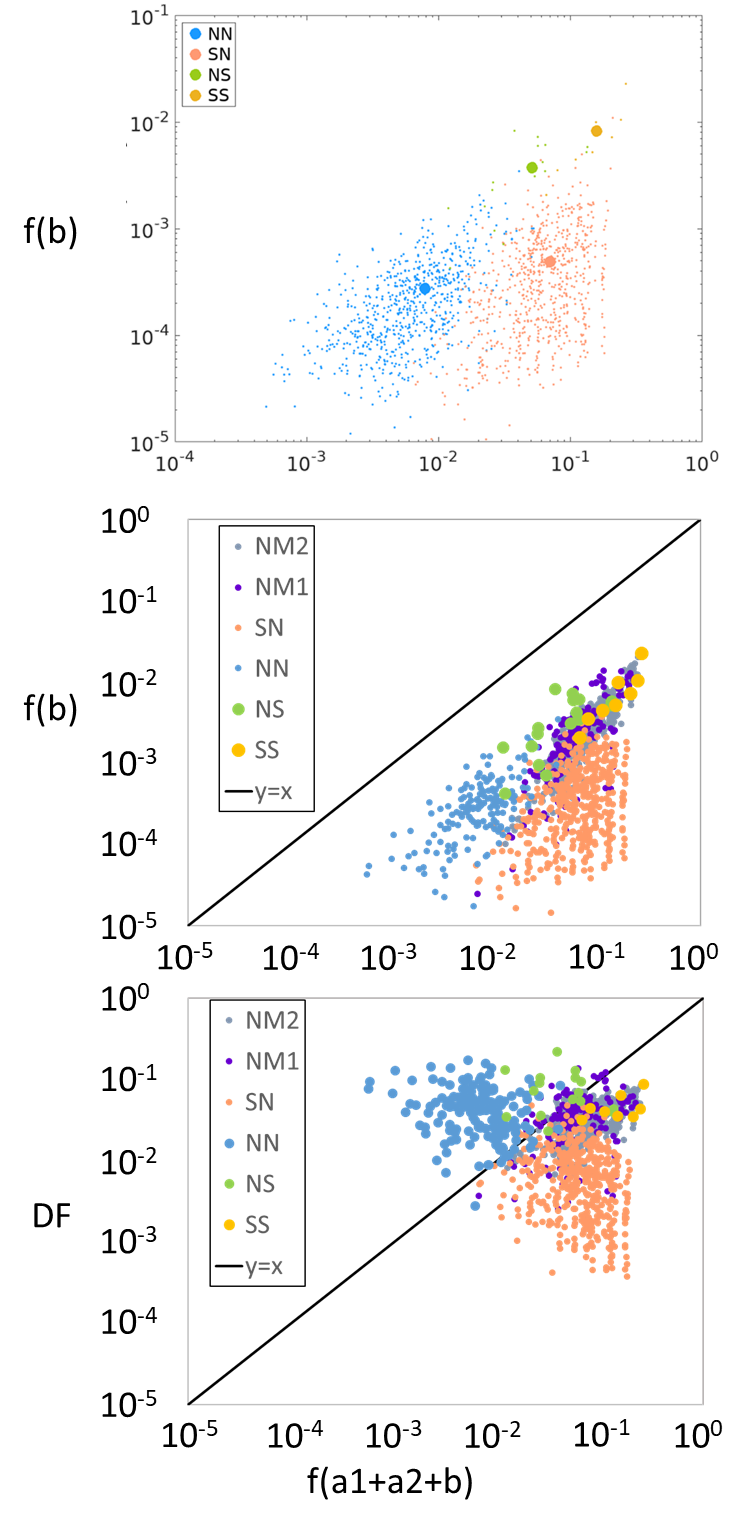


**Figure S7.** (A) Dependency of double frequency f(b) on the sum of single and double frequencies (Spearman correlation of: 0.56, 0.4, 0.97 and 0.7 for NN, SN, SS and NS respectively, with respective p-values: 6.018x10^-58^, 3.15x10^-29^, 3.96x10^-04^ and 3.23x10-^03^), and the separation of the four classes to unique locations in the double vs. single space. Large dots denote the mean single and double frequencies for each class. (B) Same as A but also including NM1 and NM2. (C) DF vs. the sum of single and double substitution frequencies.


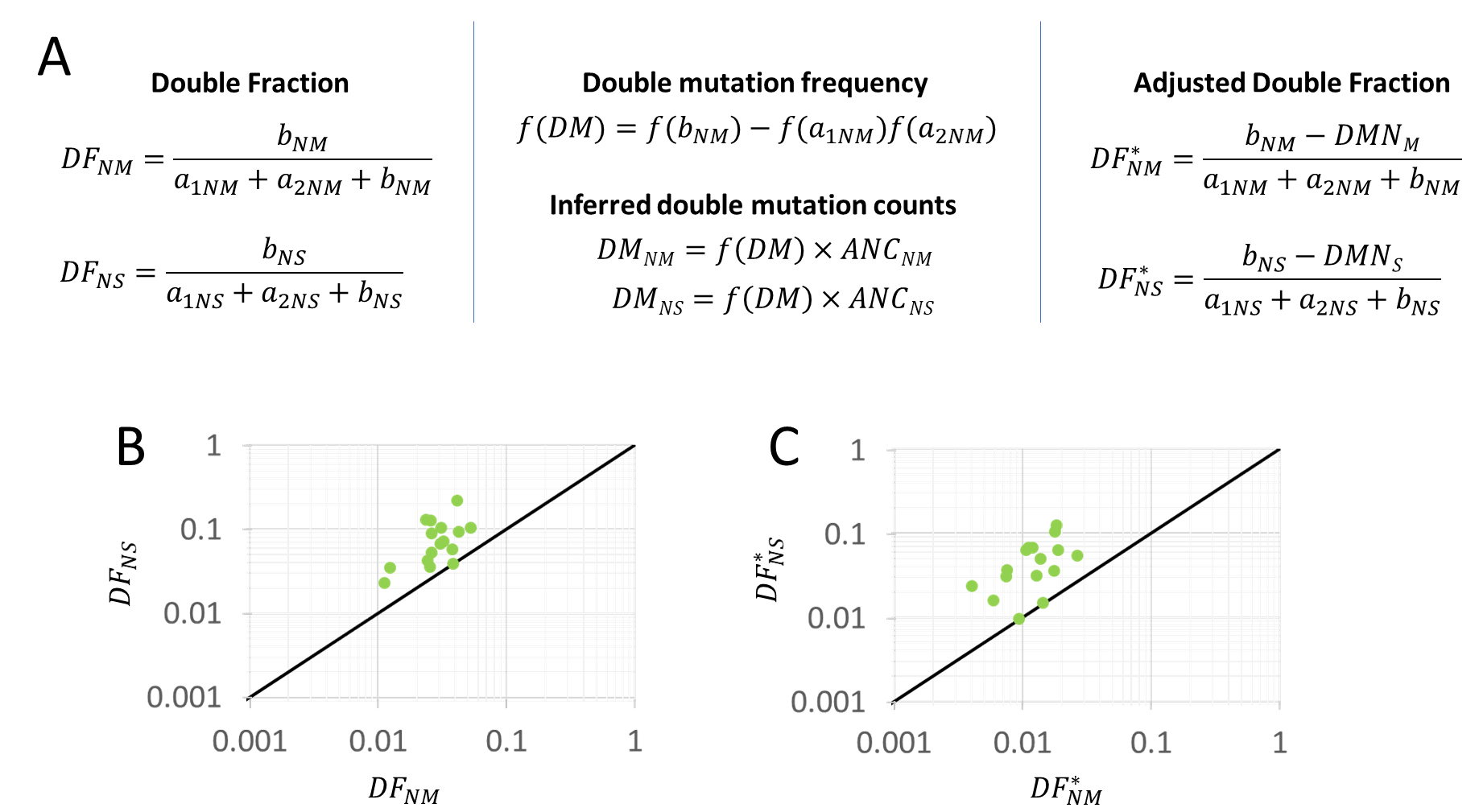


**Figure S8.** (A) DF_NM_ denotes the DF calculated for NM2 control combinations equivalent to the 16 NS combinations, denoted by DF_Ns_. Likewise, b, a1 and a2 are followed by either NM or NS to denote counts of the control combinations or the NS combinations, respectively. The frequency is denoted f() and is used to estimate the double mutation (DM) count. The DM frequency f(DM) is calculate by subtracting the product of the NM2 single synonymous substitution frequencies (frequencies of a1_NM_ and a2_NM_) from the synonymous NM2 double substitution (b_NM_). The double mutation count (DM) is then estimated separately for the control NM2 combinations, and for NS combinations by multiplying the double mutation frequency f(DM) by the count of the ancestral state of each NM2 combination and each NS combination respectively. Calculation of the adjusted DF for NM2 (DF*_NM_) and the adjusted DF for NS (DF*_Ns_) was done by subtracting the estimated double mutation counts from the observed double substitutions counts both in the NS codons and in their respective NM2 controls with the same nucleotide changes. (B) DF_NS_ vs. DF_NM_ in 16 double substitution combinations in the NS class. (C) DF*_NS_ vs. DF*_NM_ in 16 double substitution combinations in the NS class.
